# Interrelation of the CdTe Grain Size, Postgrowth Processing, and Window Layer Selection on Solar Cell Performance

**DOI:** 10.1021/acsami.2c07609

**Published:** 2022-09-09

**Authors:** Thomas P. Shalvey, Heath Bagshaw, Jonathan D. Major

**Affiliations:** † Stephenson Institute for Renewable Energy, Department of Physics, University of Liverpool, Liverpool L69 7ZF, U.K.; ‡ SEM Shared Research Facility, School of Engineering, University of Liverpool, Liverpool L69 3GL, U.K.

**Keywords:** CdTe, solar cell, grain size, interface, device performance, CdSe, SnO_2_

## Abstract

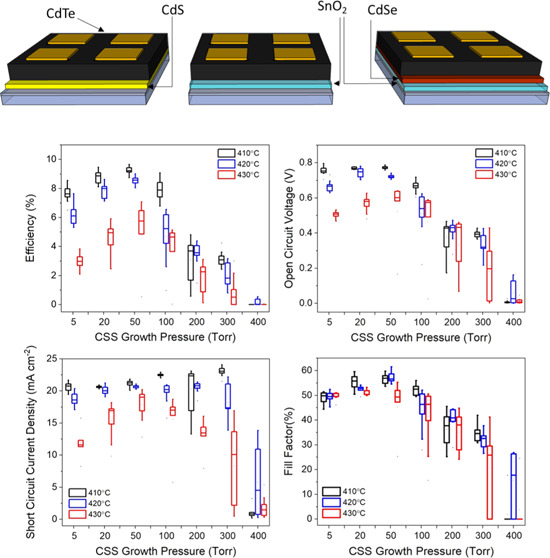

Recent improvements to the CdTe solar cell device structure have focused on replacing the CdS window layer with a more transparent material to reduce parasitic absorption and increase *J*_sc_, as well as incorporating selenium into the absorber layer to achieve a graded band gap. However, altering the CdTe device structure is nontrivial due to the interdependent nature of device processing steps. The choice of the window layer influences the grain structure of the CdTe layer, which in turn can affect the chloride treatment, which itself may contribute to intermixing between the window and absorber layers. This work studies three different device architectures in parallel, allowing for an in-depth comparison of processing conditions for CdTe solar cells grown on CdS, SnO_2_, and CdSe. Direct replacement of the CdS window layer with a wider band gap SnO_2_ layer is hindered by poor growth of the absorber, producing highly strained CdTe films and a weak junction. This is alleviated by inserting a CdSe layer between the SnO_2_ and CdTe, which improves the growth of CdTe and results in a graded CdSe_*x*_Te_1–*x*_ absorber layer. For each substrate, the CdTe deposition rate and postgrowth chloride treatment are systematically varied, highlighting the distinct processing requirements of each device structure.

## 1 Introduction

Three of the key influences on the performance of CdTe thin film solar cells are: (i) the CdTe grain structure, (ii) the chloride treatment, and (iii) the n-type “window” layer. Typically, these are treated as separate entities, but in reality, there is a distinct interrelation between them. The growth conditions of the CdTe determine the grain structure, which may require modification of the postgrowth chloride conditions, and both have an influence on interdiffusion and defect reduction with the window layer. Historically, devices have been based on a heterojunction with CdS because a homojunction is not feasible due to a strong absorption coefficient resulting in a shallow junction close to a highly defective surface.^1^ This architecture had not significantly changed for around 40 years, despite the relatively low band gap of CdS and well-established parasitic absorption which limits the available current output from devices. To alleviate these losses, several strategies have been employed to increase the amount of light reaching the CdTe layer. Alloying CdS with higher band gap materials such as ZnS allows the band gap to be varied between 2.4 and 3.6 eV, which enables some improvement in the blue response; however, this is accompanied by an increased resistivity, and for Cd_1–*x*_Zn_*x*_S compositions with band gaps above 3 eV, performance deteriorates rapidly.^2^ Simply reducing the thickness of the CdS layer can be effective to some extent, although this is limited by the requirement for a pinhole-free film that is not consumed by interdiffusion during the subsequent processing steps.^3^ The use of a high resistivity transparent (HRT) buffer layer allows for thinner CdS to be used, while maintaining *V*_oc_ and fill factor.^4^ Combining a suitable HRT layer with a nanostructured CdS:O film, which can controllably increase the CdS band gap,^5^ improves current collection, although this is also accompanied by increased series resistance.^6^ Recent insights into the importance of band alignment have allowed the CdS layer to be eliminated entirely and replaced with a ZnO layer alloyed with MgO (MZO) to vary the band gap, thereby tuning the conduction band offset to optimize transport across the interface.^4^ Depositing CdTe onto MZO substrates has enabled higher efficiencies due to increased current density, although careful process control is required to prevent a secondary barrier at the front contact from causing abnormal *JV* curves, which severely lower the fill factor.^7^ Different processing strategies have emerged among various research groups to prevent this “S” shaped curve such as limiting the layer thickness,^4^ reducing the oxygen content,^7^ varying the Mg/Zn ratio,^8^ and postgrowth annealing.^9^ This strong sensitivity to processing conditions as well as indications of degradation due to the MgO content in MZO films reacting with water vapor^10^ mean that other alternative partner layers remain worthy of investigation.

Devices with a graded CdSe_*x*_Te_1–*x*_ absorber layer forming a junction with SnO_2_ have recently shown high efficiency,^11^ demonstrating this as a suitable substrate for growing CdTe-based devices. SnO_2_ is investigated here in direct comparison to CdS as an alternative window layer for CdTe based solar cells. The widespread use of SnO_2_:F (FTO) as the front contact for CdTe PV devices means SnO_2_-based HRT layers are a natural choice, with HRT/FTO bilayers perhaps offering favorable interface properties as well as being attractive from a manufacturing perspective. Indeed, such bilayer films are already commercially available and offer a consistent substrate to develop CdTe devices.

The use of alternative window layers means that a reconsideration of device processing is necessary, and it is not sufficient to assume that optimal conditions will remain identical. Varying the window layer structure, CdTe growth conditions, and postgrowth processing in parallel, allows interdependency between the device structure and processing to be investigated. Three separate interface structures are considered (i) CdS/CdTe, (ii) SnO_2_/CdTe, and (iii) SnO_2_/CdSe/CdTe. For each structure the CdTe deposition rate is modified by variation of the close space sublimation (CSS) deposition pressure, enabling the impact on grain size and preferred orientation to be determined for each interface combination. By varying, in parallel, the postgrowth chloride annealing step, we are able to separate the impacts of growth from postgrowth processing on factors, such as interdiffusion, on device performance.

## 2 Materials and Methods

### 2.1 Materials

CdS/CdTe devices were grown on Tec15 glass substrates (NSG Ltd.) which are coated with a SnO_2_:F transparent conducting oxide (TCO) layer by the manufacturer. SnO_2_/CdTe and SnO_2_/CdSe_*x*_Te_1–*x*_ devices were grown on Tec15M glass substrates, which are identical to Tec15 except for the presence of an additional 100 nm, nominally undoped SnO_2_ layer on top of the TCO. CdS and CdSe layers were deposited via sputtering in 5 mTorr Ar, with a substrate temperature of 200 °C and power density of 1.32 W cm^–2^.

CdTe was deposited onto 100 nm films of CdS, SnO_2_, or CdSe via CSS in a custom-built, all-quartz deposition system.^12^ Polycrystalline lumps of CdTe (Alfa Aesar, 5N) were placed into the source tray, with the 5 × 5 cm^2^ substrate positioned ∼8 mm above. The source and substrate temperatures were maintained at 650 and 550 °C, respectively, and the process was carried out under a N_2_ ambient at pressures between 5 and 400 Torr. Varying the CSS deposition pressure affects the adatom arrival rate at the substrate surface,^12^ controlling the CdTe deposition rate and nucleation kinetics.^13^

**Figure 1:**
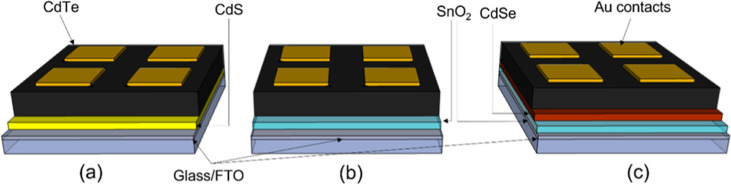
Diagrams showing the three device architectures used in the study, whereby CdTe is deposited onto (a) 100 nm CdS, (b) 100 nm SnO_2_, or (c) 100 nm SnO_2_ and 100 nm CdSe bilayer. Note the CdSe layer interdiffuses with CdTe during CSS deposition to form a CdSe_*x*_Te_1–*x*_ layer.

To ensure a consistent film thickness while using different deposition pressures, the growth duration was varied between 4 and 162 min, resulting in a ∼7 μm absorber layer for all samples. See Table S1 for further details. This relatively thick absorber layer minimizes the risk of pinholes and the number of shunted devices on each sample plate, albeit at the expense of increased series resistance. Where CdTe is deposited onto CdSe, the interdiffusion that occurs during the high-temperature growth process results in a CdSe_*x*_Te_1–*x*_ absorber layer.^14^ The CdS/CdTe, SnO_2_/CdTe, and SnO_2_/CdSe_*x*_Te_1–*x*_ device structures are shown in Figure 1.

To make photovoltaic devices, each of these film stacks was subjected to a MgCl_2_ activation treatment for 20 min at temperatures between 410 and 430 °C. Samples were submerged in a dilute nitric-phosphoric acid etch for 15 s both before and after MgCl_2_ treatment. A 50 nm Au layer was then evaporated onto the back surface through a 0.25 cm^–2^ mask to define nine cells per device. No intentional Cu-doping process was included as devices perform at a good level without it, and this was viewed as an additional variable that could easily be removed. It is worth noting this does, however, reduce the peak of performance.

## 2.2 Methods

Electron microscopy (SEM) was performed using a JEOL 6610 and JEOL 7001 FEG SEM. The grain size was determined by manual tracing of grain boundaries, and the grain area was extracted using ImageJ for ∼200 grains per sample. This is then presented as an average grain radius assuming circular grains with an equivalent area. X-ray diffraction (XRD) measurements were taken using a Rigaku SmartLab diffractometer with a Cu rotating anode (λ = 1.542 Å) and a Ge(220) × 2 monochromator in parallel beam configuration. The texture coefficients for the diffraction peaks were determined by comparing the measured diffraction intensity to that expected for powdered samples using the Harris method.^15^ The standard deviation of the texture coefficient for all measured peaks then gives an indication of the extent to which a film is preferentially oriented. A backlit optical microscope was used to observe pinhole formation, and the pinhole fractional area was determined by averaging the above-band-gap light transmission (500–800 nm) through optically thick (∼7 μm) films using a Shimadzu SolidSpec-3700 UV–vis spectrophotometer.

Current density–voltage (*JV*) measurements were taken under AM1.5G illumination using a TS Space Systems solar simulator (class AAA). A Bentham PVE300 measurement system was used to record the external quantum efficiency (EQE). A Solartron SI1260 impedance analyzer was used for capacitance–voltage (CV) profiling with a DC bias voltage between −0.5 and +0.5 V and 30 mV AC perturbation voltage.

## 3 Results

### 3.1 CdS/CdTe Devices

**Figure 2:**
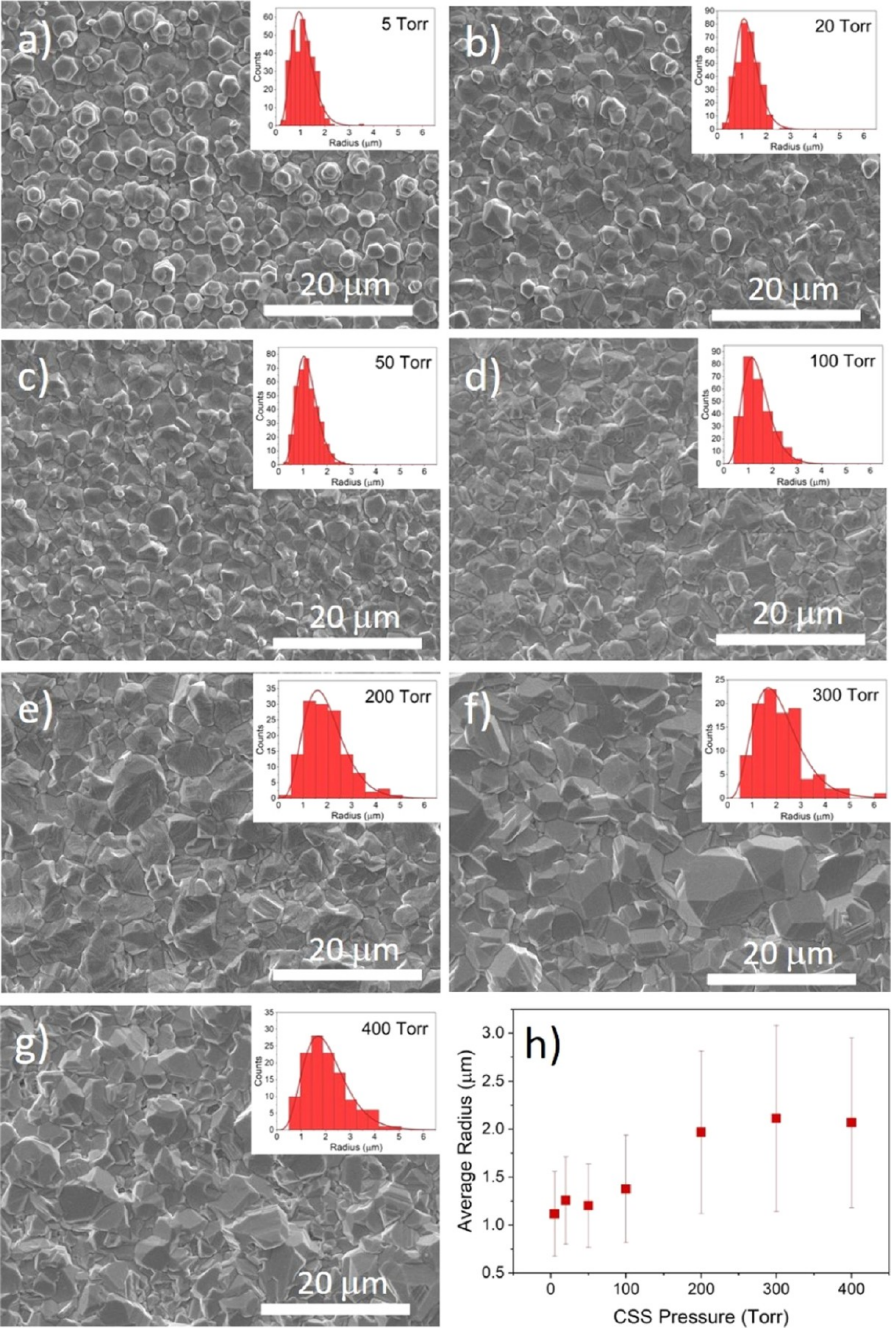
SEM images of the back surface of as-grown CdTe films deposited on CdS at pressures between 5 and 400 Torr (a–g) with the grain size distribution shown in the inset and the mean radius plotted as a function of deposition pressure (h).

**Figure 3:**
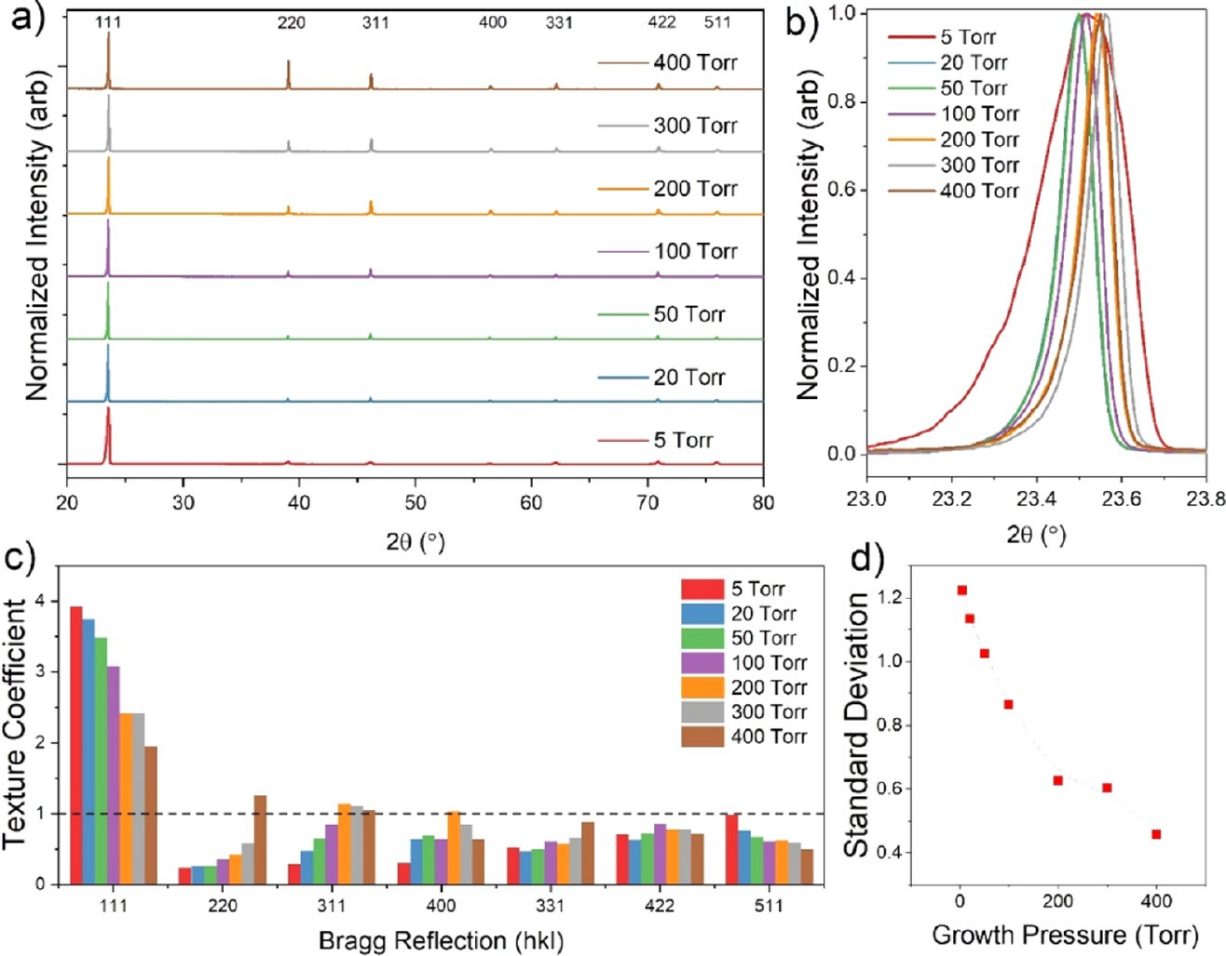
XRD data for 7 μm CdTe films grown on CdS-coated substrates under varying pressure of nitrogen (a), with higher magnification of the 111 peak shown in (b). The texture coefficient for each Bragg reflection at each growth pressure is given in (c) and their standard deviation in (d).

Figure 2 shows SEM images of as-grown CdTe films deposited on a CdS window layer at pressures between 5 and 400 Torr (a–g), as well as the average grain radius plotted as a function of deposition pressure (h). The figure also shows histograms of the grain sizes each fitted to a gamma distribution. For low growth pressures, especially 5 Torr, there are distinct hexagonal crystal facets consistent with (111) planes bounded by ⟨110⟩ directions at their six edges. This is consistent with the close-packed (111) planes dominating the preferred orientation, as is shown in XRD measurements in Figure 3. As the pressure increases, the grain shape becomes more irregular as other orientations become more prominent while the grains increase in size. The grain size initially follows a symmetric, narrow distribution at low pressures and becomes skewed toward larger grains at higher growth pressures. The average grain radius shown in Figure 2h increases with growth pressure before leveling out above 300 Torr.

This demonstrates that the CdTe grain size can be effectively controlled by altering the nucleation conditions via nitrogen pressure. Increasing the nitrogen growth pressure reduces the adatom arrival rate of Cd and Te_2_ species at the substrate, thereby reducing the nucleation density of stable islands. These well-spaced islands can then grow uninterrupted as the film develops, resulting in large grain sizes.^12,13^

Figure 3a shows θ–2θ XRD spectra for these films, which are strongly [111] oriented, especially samples grown at lower pressures. The 5 Torr samples show only minor contributions from other peaks, which is consistent with observations from Figure 2a. The 111 peak for each growth pressure is shown at higher magnification in Figure 3b, where there is a clear asymmetry in peak shape for all samples. This is most apparent for the sample deposited at 5 Torr, whereby the 111 peak is significantly broader than for other samples, with a widened shoulder toward the low angle side of the peak. Several mechanisms could possibly contribute to this asymmetric peak such as crystallite size, lattice strain, and intermixing of the CdS and CdTe layers. However, because peak broadening due to crystallite size is dominant for much smaller (<100 nm) grain sizes than those involved here, and sulfur interdiffusion would be expected to decrease the lattice spacing and therefore increase the diffraction angle, inhomogeneous lattice strain appears to be the most likely cause of the observed peak broadening. In-plane compressive strain is expected at the CdS–CdTe interface due to the smaller lattice constant of CdS, which increases the lattice spacing parallel to the interface according to Poisson’s ratio. Therefore, this region of increased lattice spacing could contribute to the low-angle shoulder of the 111 peaks. This would be most apparent for low-pressure growth, whereby rapid deposition and less intermixing maintain this interfacial strain, causing the enhanced broadening seen for the 5 Torr sample. The peak shape for higher growth pressures is more consistent, with no change in FWHM and less obvious asymmetry. The peak maxima follow a pattern of increasing diffraction angle with growth pressure, with a small reversal of the trend for 5 and 400 Torr. This indicates a decrease in lattice constant from 6.55 to 6.53 Å, which may be a result of sulfur diffusion^3^ or a small relaxation of lattice strain.

Figure 3c shows the texture coefficient for each Bragg direction as a function of deposition pressure, whereby a texture coefficient of 1 corresponds to the peak intensity expected for a randomly oriented film with an equal distribution of all possible crystal orientations. At higher growth pressures, the reflections from crystallographic planes other than 111 and 511 gradually increase, indicating a more randomized texture. The intensities from the 220, 311, and 331 planes increase as the preferential orientation of the 111 plane is reduced. In contrast, the 400 and 422 orientations appear to be relatively insensitive to the growth pressure showing no trend while the texture coefficient for the 511 orientation decreases, with progressively lower counts than would be expected for a powder diffraction pattern. This is presumably because {511} is the Σ = 3 twin orientation to {111} in the zinc-blende lattice; therefore, the 511 intensity follows the same decreasing trend as the 111 peak intensity.^16^ Despite this, the standard deviation decreases rapidly with growth pressure indicating a more randomized texture, as shown in Figure 3d. This is dominated by a decrease in the 111 texture coefficient and can be attributed to the change in growth pressure altering the nucleation density during the early stage of CdTe film formation.^13^ Islands nucleate with random orientations, with dense crystallographic orientations such as the (111) plane growing quicker than others. At low pressure, the high density of nucleation sites means the larger (111) orientated islands interact with and outgrow the slower growing orientations, resulting in a strong preferential orientation. Higher growth pressure lowers the nucleation density, thereby limiting interactions between islands and allowing the original random orientation of the islands to be preserved during film growth.

**Figure 4:**
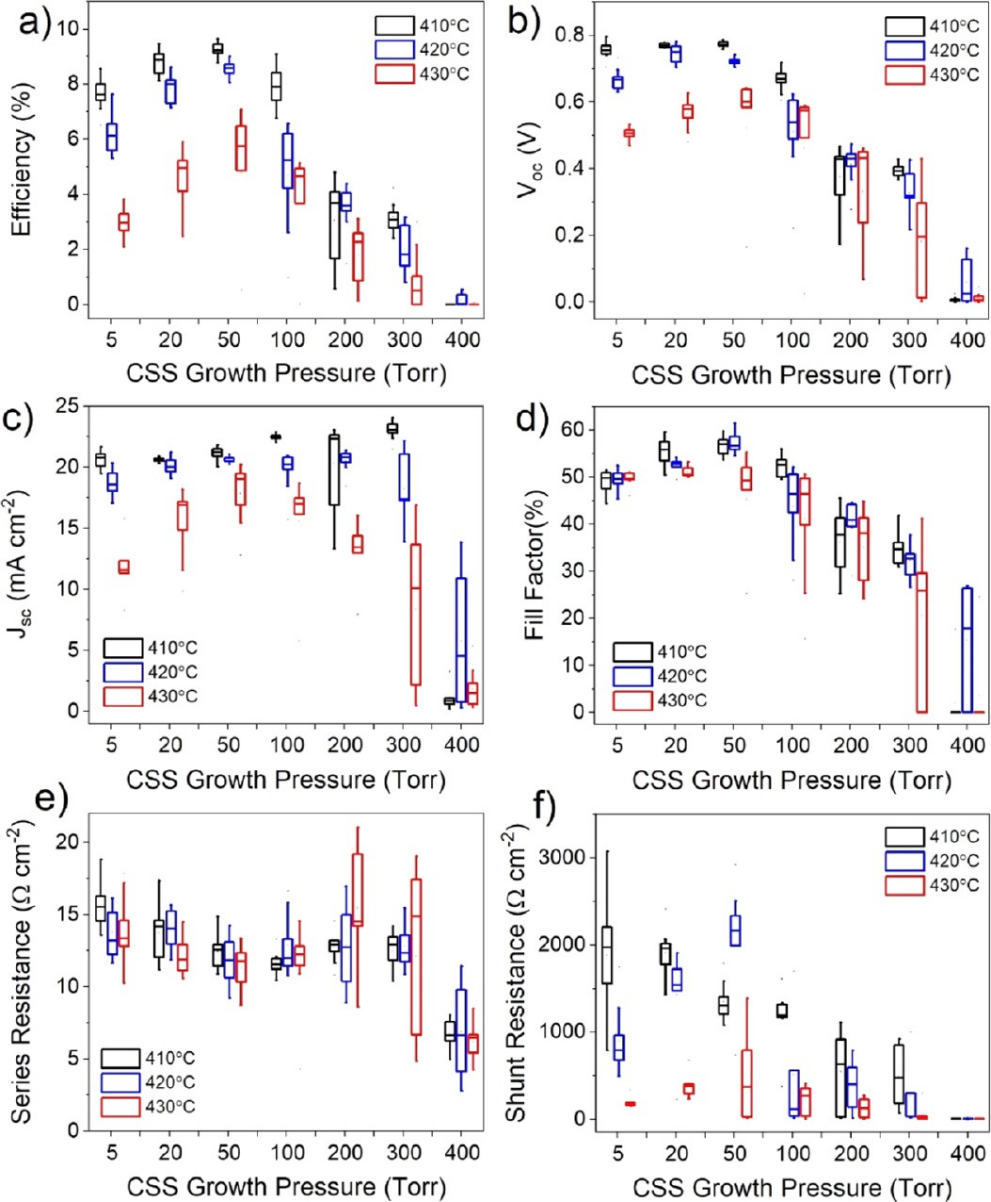
Box and whisker plots showing *JV* performance parameters for CdS/CdTe devices grown under 5–400 Torr of nitrogen, and activated at 410, 420, and 430 °C for each growth pressure. The box boundaries show the upper and lower quartiles with a horizontal line for the median value, and the range given by the whiskers. The efficiency (a), open circuit voltage (b), short circuit current density (c), fill factor (d), series resistance (e), and shunt resistance (f) are given as a function of growth pressure.

A series of solar cells were processed from the films grown with the same series of varied CdTe deposition pressure. Because device efficiency is highly sensitive to temperature during the activation treatment and Cl diffusion is grain boundary dominated,^17^ the MgCl_2_ temperature was also varied between 410 and 430 °C for each deposition pressure. The results are given in Figure 4, which shows box plots of the performance parameters from devices with nine cells for each combination of growth pressure and MgCl_2_ temperature. *JV* curves for the highest performing contacts in each device are shown in Figure 4. The efficiency of devices gradually increases with higher pressure before showing a clear peak for all treatment temperatures at 50 Torr, after which performance deteriorates rapidly. Devices grown at 400 Torr show virtually no photovoltaic performance and only a very weak diode response. At 410 °C, the open circuit voltage (*V*_oc_) is not significantly changed for growth pressures up to 50 Torr after which it begins to decrease; however, for 420 and 430 °C, there is more of a *V*_oc_ dependence on growth pressure below 50 Torr. Similarly, the short circuit current density (*J*_sc_) is not detrimentally affected by growth pressure for the 410 °C series, excluding the 400 Torr sample. Instead, there is a small increase in *J*_sc_ at higher pressure due to a thinner CdS layer which is consumed during longer growth runs. At the higher activation temperature of 430 °C, the devices appear overtreated, showing lower efficiencies overall and a stronger dependence on growth pressure which peaks at 50 Torr. The series resistance initially decreases with growth pressure which could be due to the modest increase in grain size resulting in fewer grain boundaries or could be due to better interfacial properties with longer depositions that accompany the high-pressure growth. However, because the series resistance begins to increase at pressures >100 Torr, despite the larger grain size for these films as shown in Figure 2h, the grain size is not expected to be the dominant effect. Shunt resistance is decreased significantly for high growth pressures, as well as for 430 °C activation treatments. This could be due to the consumption of the CdS layer during growth, meaning that there is effectively no n-type partner layer to form a heterojunction with, or due to an increased surface roughness, which is expected to accompany a larger grain size, resulting in thinner absorber areas leading to pathways for current to short circuit the device.

**Figure 5:**
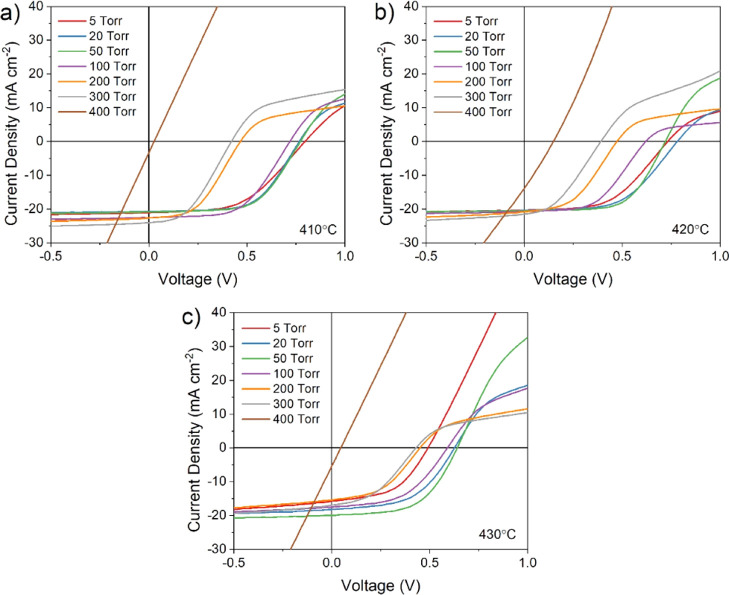
*JV* curves for the highest efficiency contact of CdS/CdTe devices grown under varied N_2_ pressure and treated at (a) 410, (b) 420, and (c) 430 °C.

Figure 5 shows *JV* curves corresponding to the highest efficiency contact from the series shown in Figure 4, comparing the effect of CdTe growth pressure on devices treated at 410, 420, and 430 °C. For all treatment temperatures, there is a clear difference between the shape of *JV* curves for devices grown at low and high pressure. Low-pressure growth (i.e., below ∼100 Torr) results in a typical *JV* response for CdTe devices, with a clear turn-on voltage, open circuit voltage between 0.7 and 0.8 V, and rollover at a higher forward bias due to the effect of a back contact barrier. Rollover is expected for these devices, which have no intentional copper-doped region or other contact layers that would otherwise reduce the barrier height in an effort to minimize process variables. As the growth pressure is increased above 100 Torr, a rapid drop in *V*_oc_ is accompanied by a much more severe rollover effect. This is likely due to a deterioration of the CdS–CdTe junction either due to intermixing of the two layers during the longer growth duration required for high-pressure growth, or the resulting large grain structure offering leakage paths due to increased surface roughness. In any case, a weakened CdS–CdTe junction will be more susceptible to rollover, as the back contact barrier will dominate the main junction at a lower forward bias, therefore showing enhanced current blocking behavior. For devices grown at 400 Torr, the *JV* response is almost entirely linear, showing no diode-like behavior for all MgCl_2_ treatment temperatures and barely entering the fourth quadrant in which power can be extracted from the solar cell. This near Ohmic response indicates a very poor junction quality which is not able to effectively separate photogenerated carriers, with the solar cell instead acting largely as a resistor.

**Figure 6:**
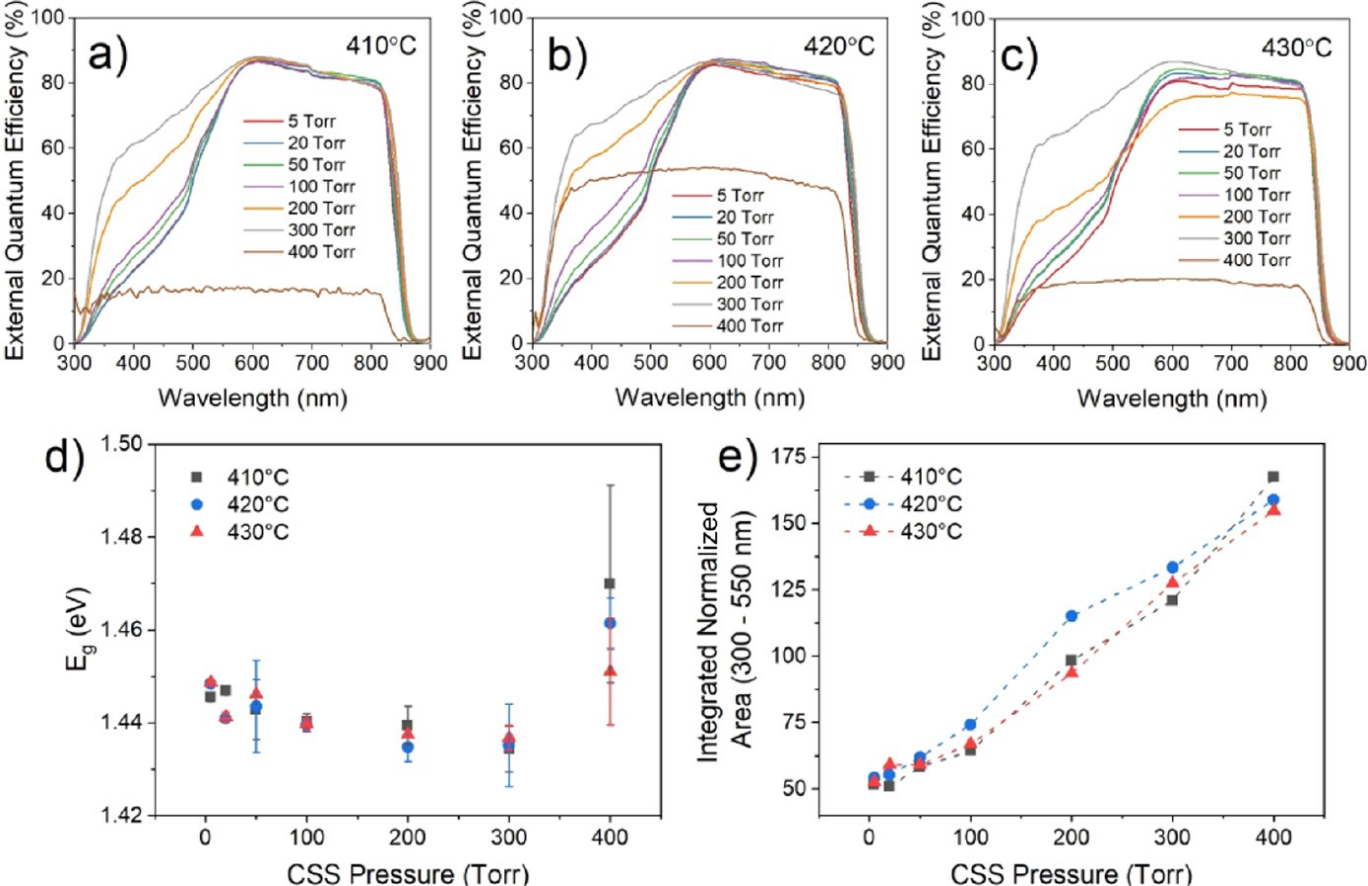
EQE spectra for devices grown on CdS under 5–400 Torr nitrogen and subject to MgCl_2_ treatment at 410 (a), 420 (b), and 430 °C (c). The minimum absorber band gap taken from linear extrapolation of the CdTe absorption edge (d) and integrated area of these EQE curves in the region of 300–550 nm after normalizing to the point of maximum collection efficiency (e) are shown as a function of growth pressure.

The mechanism behind the changes in performance as a result of grain size and postgrowth treatment can be interrogated via EQE measurement. Figure 6a–c shows EQE curves for the highest efficiency contact from the CdS/CdTe devices described previously, grown under 5–400 Torr N_2_ and treated with MgCl_2_ at temperatures between 410 and 430 °C. The minimum absorber band gap is determined from these EQE curves by extrapolating the linear section of the long wavelength cut-off region and is plotted as a function of growth pressure for each activation temperature in Figure 6d. By normalizing the EQE curves to the point of maximum collection efficiency and comparing the region 300–550 nm, an assessment of the blue response can be determined without the influence of differences in overall collection efficiency. This is accomplished in Figure 6e, which shows the area under the short wavelength region of these normalized EQE curves as a function of growth pressure.

The short wavelength region (300–550 nm) is dominated by parasitic light absorption in the CdS layer, which absorbs light but does not contribute to photocurrent because carriers are not collected efficiently.^6^ Therefore, samples deposited at higher pressure, in which the thickness of the remaining CdS layer has been reduced by interdiffusion during long growth durations, show a comparatively higher EQE response in this region. The opposite applies for low deposition pressures, whereby short growth durations lead to limited interdiffusion and therefore thicker CdS, resulting in a characteristic shoulder in the short wavelength EQE response. The shoulder region (∼500–550 nm), therefore, corresponds to the degree of interdiffusion between the CdS and CdTe layers. Devices grown at 400 Torr show a significantly reduced EQE response across all wavelengths due to poor junction quality; therefore, the efficiency is lower in the short wavelength region compared to other devices. There is no indication of parasitic CdS absorption, implying that the CdS layer has been completely consumed by intermixing and likely accounts for the poor device performance. Normalizing the EQE curves allows the shape of the response to be compared directly instead of the absolute magnitude. This is shown in Figure 6e as a function of growth pressure for each MgCl_2_ treatment temperature. The area under the short wavelength region of normalized EQE curves increases linearly with growth pressure, corresponding to a gradual increase in the extent of intermixing between the CdS and CdTe layers as the growth duration is increased. This does not vary with MgCl_2_ treatment temperature, which confirms observations by Taylor et al.^19^ that CdS–CdTe intermixing for CSS-grown devices occurs primarily during CdTe deposition rather than chlorine activation.

The maximum EQE response occurs around 600 nm, with a small, gradual reduction in quantum efficiency at longer wavelengths due to deeper penetration into the CdTe layer, meaning photogenerated carriers are produced further from the junction at which they are separated. For each activation temperature, all devices show a similar response with no systematic change in growth pressure. However, devices that are grown at the same pressure but undergo MgCl_2_ treatment at different temperatures show subtle differences. Higher MgCl_2_ temperature is correlated with a flatter gradient, indicating better collection further into the device. This is likely due to the increased depletion width for high activation temperatures as a result of lower doping density (see Figure S1).

The long wavelength cut-off region of the EQE curves is dominated by the minimum band gap of the absorber layer, which comprises an intermixed CdS_*y*_Te_1–*y*_ phase^20^ for these devices and is therefore expected to vary between samples according to the degree of interdiffusion. Figure 6d shows that the minimum absorber band gap decreases with increasing growth pressures up to 300 Torr, irrespective of MgCl_2_ treatment temperature. The extended growth durations which accompany higher pressure growth result in more interdiffusion of the CdS and CdTe layers, with dilute sulfur compositions reducing the band gap of CdS_*y*_Te_1–*y*_ compared to CdTe via the bowing effect.^20^ At 400 Torr, there is a reversal of this trend whereby the absorber band gap increases. As interdiffusion continues and the phase becomes more sulfur rich, the band gap of CdS_*y*_Te_1–*y*_ increases toward that of CdS. The lack of dependence on activation temperature again confirms that interdiffusion occurs primarily during deposition.

The efficiency of CdS/CdTe devices has been shown to be highly sensitive to both the activation treatment and growth pressure. Higher CSS growth pressures, and therefore slower deposition rates, increase the CdTe grain size and produces a more random texture. While this would imply high growth pressures should be optimal for device performance due to fewer grain boundaries, this also leads to intermixing of the CdTe and CdS layers, as evidenced by EQE measurement, resulting in a poor diode. Devices also show a significant decrease in efficiency for only a 20 °C increase in the chlorine activation temperature due to overtreatment. Although a larger grain size might be expected to inhibit grain boundary-assisted transport of chlorine to the front interface, no evidence of this is observed here. Shunt resistance appears to be most strongly affected by overtreatment in this case, likely a result of excess chlorine at the CdS–CdTe interface having a detrimental effect on the heterojunction. The CdS–CdTe device structure, therefore, limits both the MgCl_2_ treatment temperature as well as the growth duration, with deterioration of performance in both cases outside of a small window of processing conditions.

### 3.2 SnO_2_/CdTe Devices

In the previous section, high-temperature growth of CdTe films onto CdS substrates was shown to result in the intermixing and, in severe cases, consumption of the CdS window layer, limiting the thermal budget available during the processing of this device structure and limiting the achievable grain size. Furthermore, the MgCl_2_ activation temperature was restricted to 410 °C to prevent overtreatment, which reduced the net acceptor density and results in lower efficiency. A SnO_2_/CdTe device may be more tolerant to aggressive processing conditions compared to the more typical CdS/CdTe structure due to the chemically stable nature of the window layer. With this in mind, CdTe films are grown directly onto SnO_2_-coated substrates to explore any potential new parameter space offered by the more robust window layer, with the intention of maximizing grain size.

**Figure 7:**
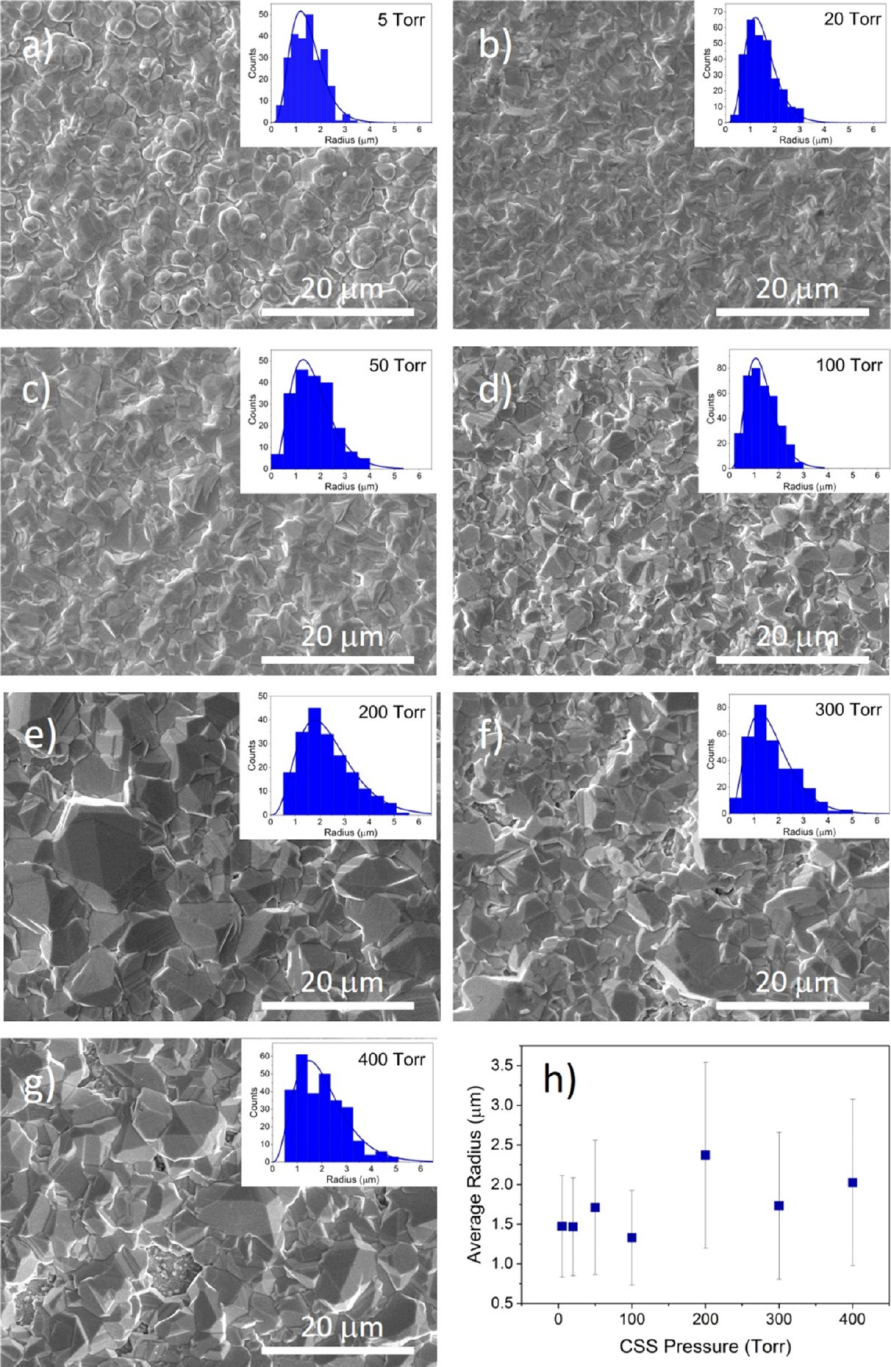
SEM images of the back surface of as-grown CdTe films deposited on SnO_2_ at pressures between 5 and 400 Torr (a–g) with the grain size distribution shown in the inset and the mean radius plotted as a function of deposition pressure (h).

Figure 7a–g shows SEM images of the back surface of as-deposited CdTe films grown directly onto SnO_2_ coated glass substrates at pressures between 5 and 400 Torr, and the average grain radius is shown as a function of growth pressure in Figure 7h. The film grown at 5 Torr (Figure 7a) shows a rounded grain structure with less pronounced hexagonal facets than previously observed for growth on CdS substrates (Figure 2a), suggesting a reduction in the extent of [111] texturing. Grains become less rounded and more irregularly shaped as the growth pressure is increased to 20 and 50 Torr, and well-defined crystal facets become clearer for growth pressures above 100 Torr. While higher pressure growth leads to a visibly larger grain structure, this is not obvious from the average grain size shown in Figure 7h, which is heavily scattered. Instead, the maximum grain size increases with growth pressure, which results in more skewed histograms, but the average remains dominated by the presence of many smaller grains. It is noted that there are large uncertainties associated with the average grain size taken from the measurement of manually defined grains, where grain boundaries are not clearly distinguished, and alternative techniques, such as EBSD, may allow for a much more accurate assessment. However, it is clear from these results that the dependence of grain size on growth pressure is much weaker for CdTe films grown on SnO_2_ substrates compared to the CdS substrates shown in Figure 2.

**Figure 8:**
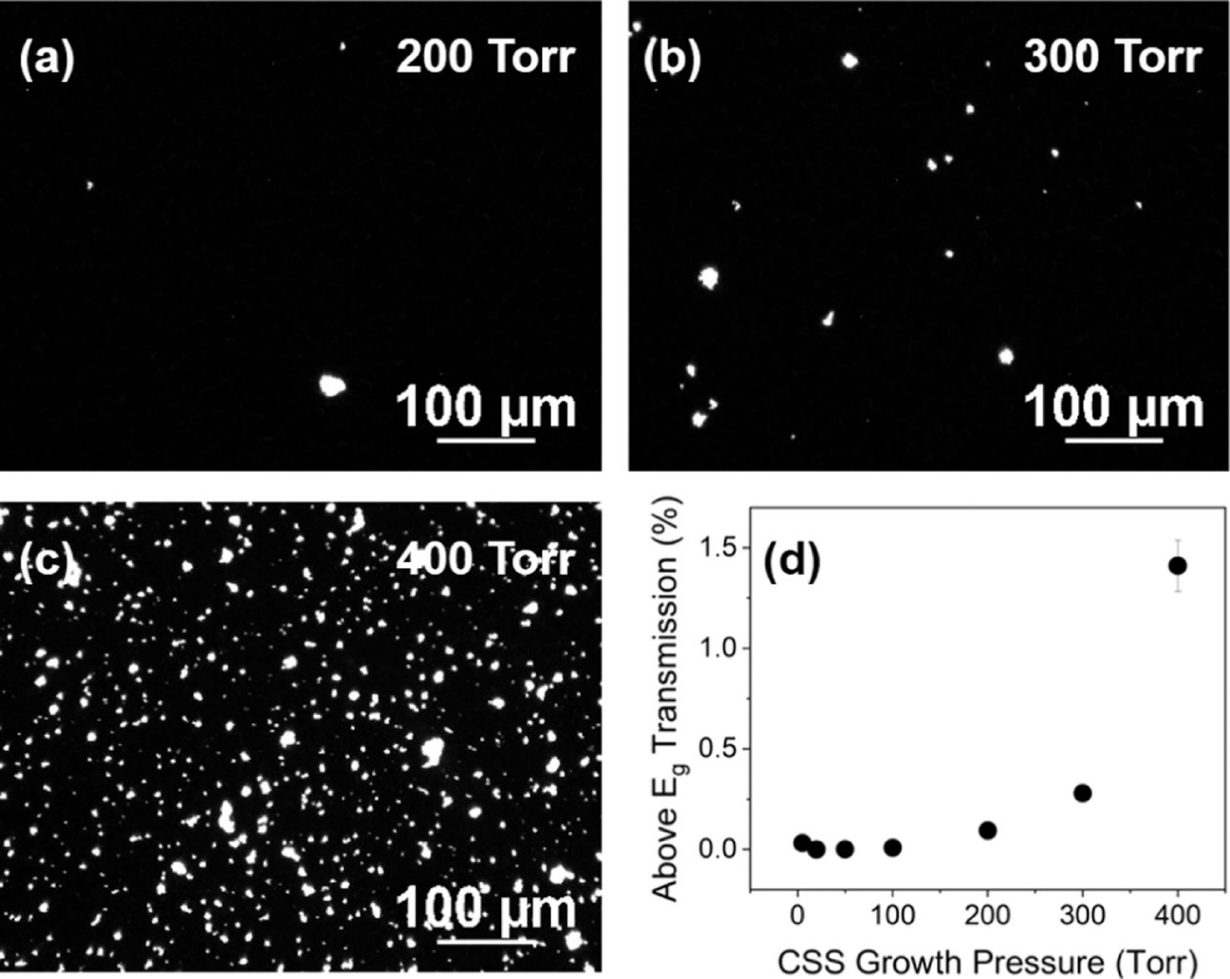
Backlit optical microscope images showing the pinhole density for SnO_2_/CdTe films grown at (a) 200, (b) 300, (c) 400 Torr, and (d) percentage of above-band-gap light transmitted through samples of CdTe on SnO_2_ substrates prior to MgCl_2_ treatment.

However, low growth pressures result in a compact, continuous CdTe film, and higher growth pressures (i.e., Figure 7f,g) result in large areas of exposed SnO_2_ substrate, which was not observed when deposited onto CdS. This is exacerbated as the growth pressure is increased and can be observed on a wider scale using backlit optical microscopy shown in Figure 8a–c. Here, the sample is illuminated from behind the device; therefore, bright areas correspond to regions of poor CdTe coverage, resulting in pinholes and direct contact between the SnO_2_ and Au in a full device structure. No pinholes were observed by optical microscopy for growth pressures up to 100 Torr; however, the pinhole density is seen to increase rapidly above 200 Torr. To quantify the pinhole area, the as-grown films were illuminated with above-band-gap light between 500 and 800 nm, which should be almost entirely absorbed by the ∼7 μm thick CdTe layer. In this way the fractional pinhole area could be estimated by the average light transmission, which is given in Figure 8d and gradually increases with higher deposition pressures.

**Figure 9:**
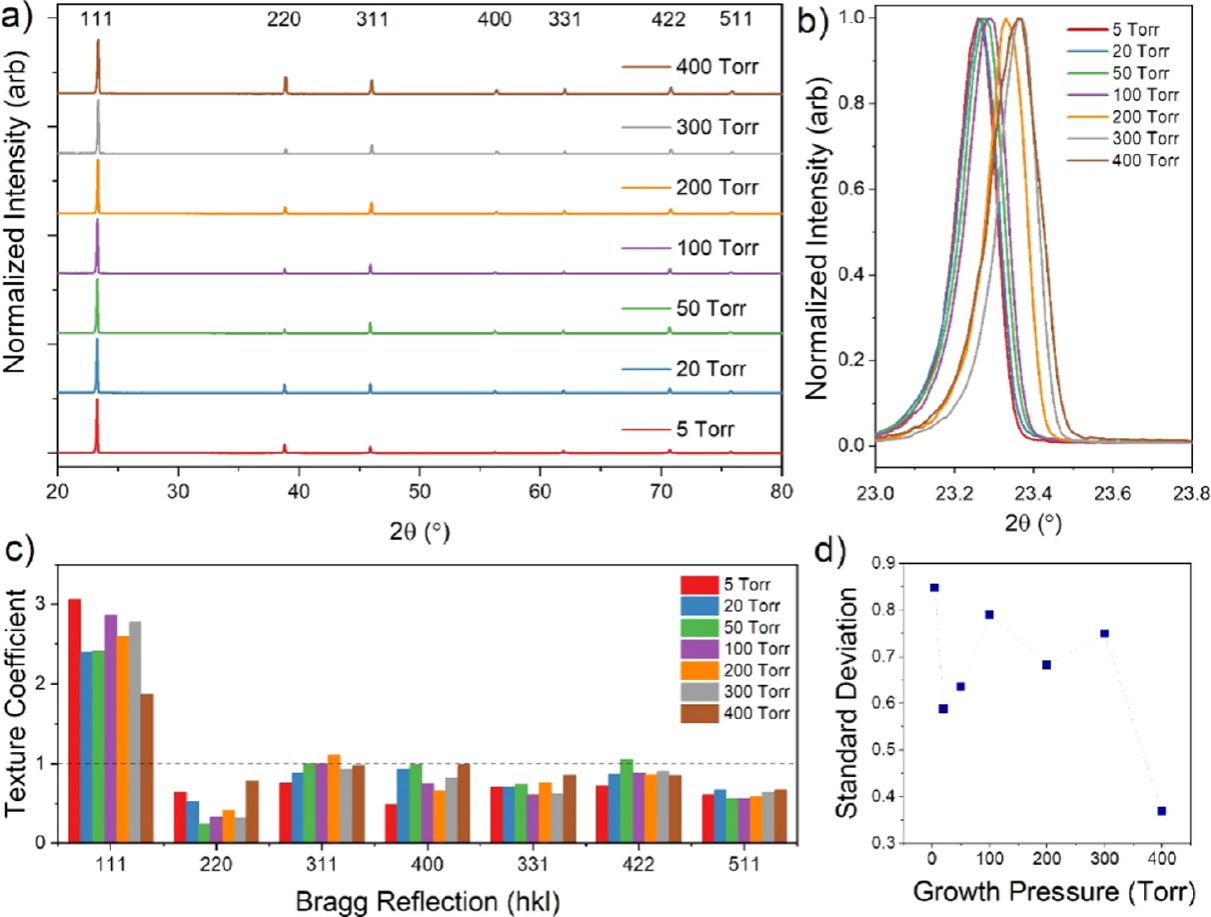
XRD data for 7 μm CdTe films grown on SnO_2_-coated substrates under varying pressure of nitrogen (a) and with higher magnification of the 111 peak shown in (b). The texture coefficient for each Bragg reflection at each growth pressure is given in (c), and the standard deviation of the texture coefficient for each sample is in (d).

Figure 9 shows XRD data for the as-grown CdTe films deposited on SnO_2_, under varying nitrogen pressure, whereby increased pressure results in longer growth times to maintain constant thickness (Table S1). The normalized diffraction patterns in Figure 9a show that the 111 peak is dominant for all samples, which is shown at higher magnification in Figure 9b. There is no variation in peak shape in contrast to films deposited on CdS; however, there is a pronounced change in peak position. This gradual peak shift indicates that the lattice constant (*a*_0_) decreases linearly from 6.62 to 6.59 Å as deposition pressure increases from 5 to 300 Torr, with no further change for the 400 Torr sample. In comparison, the expected lattice constant for a powdered (unstrained) CdTe sample is 6.48 Å,^21^ which is significantly smaller than calculated for all samples here. The larger lattice constant for these films, compared to both powdered CdTe and films grown on CdS, suggests there is more strain present in these films, which could be a result of growth on a highly lattice-mismatched substrate.^22^ Whereas interdiffusion between CdS and CdTe layers is typically relied on to relax the 10% lattice mismatch in CdS/CdTe devices,^23^ the absence of a CdS layer in these samples means the strain at the SnO_2_/CdTe interface is retained despite the prolonged high-temperature growth. The significant change in lattice constant indicated by back surface measurements of ∼7 μm thick CdTe films suggest the variation in growth conditions is having an impact on the bulk CdTe. This is likely an effect of the longer growth times for higher pressure depositions causing film relaxation at the interface, which is consistent with a gradual decrease in lattice constant toward the unstrained bulk value.

**Figure 10:**
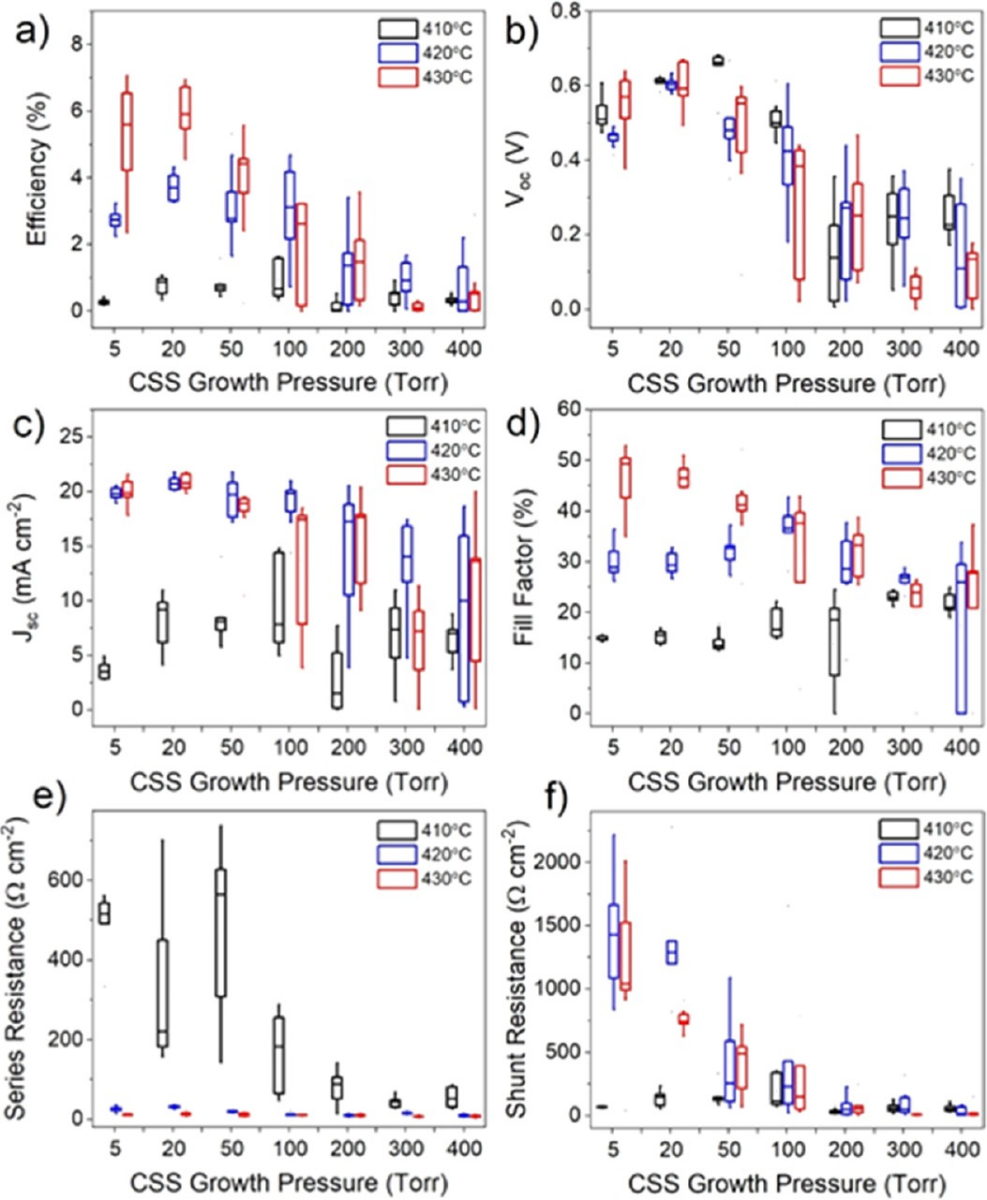
Box and whisker plots showing *JV* performance parameters for SnO_2_/CdTe devices grown under 5–400 Torr of nitrogen, and activated at 410, 420, and 430 °C for each growth pressure. The box boundaries show the upper and lower quartiles with a horizontal line for the median value, and the range given is by the whiskers. Efficiency (a), open circuit voltage (b), short circuit current density (c), fill factor (d), series resistance (e), and shunt resistance (f) are given as a function of growth pressure.

**Figure 11:**
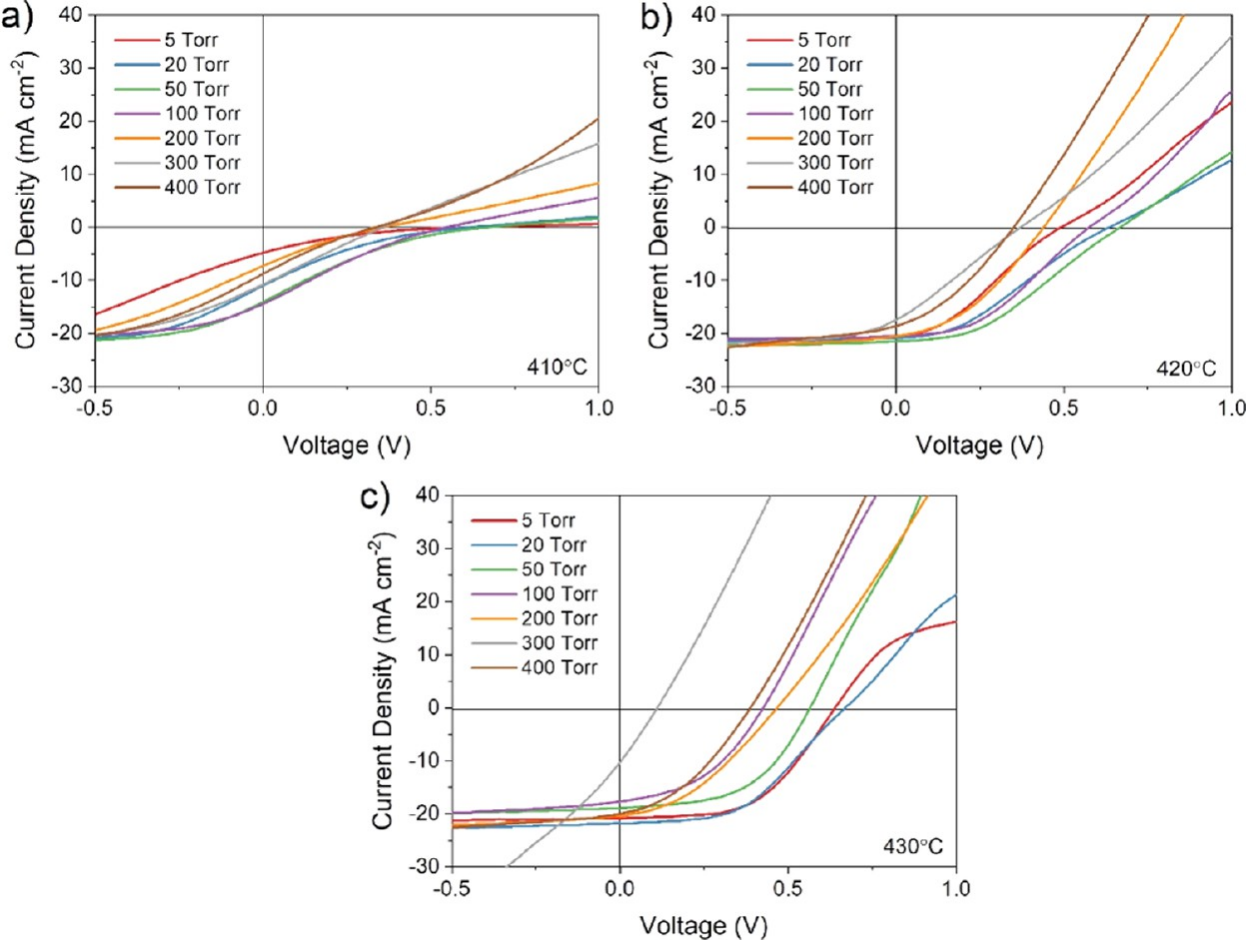
*JV* curves for the highest efficiency contact of CdTe/SnO_2_ devices grown under varied N_2_ pressure and treated at 410 (a), 420 (b), and 430 °C (c).

Figure 9c,d shows the randomization of texture with growth pressure that is observed on CdS substrates is not observed for SnO_2_. All films display a [111] preferred orientation. However, this does not change systematically with growth duration, and no trend was observed as a function of nitrogen pressure in either the texture coefficients or their standard deviation. CdS is reported to template the growth of CdTe in the [111] direction,^24^ and therefore, the use of SnO_2_ as a substrate will naturally result in fewer [111] oriented islands during the nucleation stage of CdTe deposition. This can be seen by comparing the diffraction patterns of CdTe grown at 5 Torr on CdS and SnO_2_ substrates whereby low-pressure growth on CdS results in almost exclusive [111] orientation with very small signals from the other reflections (Figure 3). In contrast, Figure 9 shows that while CdTe grown on SnO_2_ substrates retains a [111] preferential orientation, there are also relatively strong signals corresponding to reflections from several orientations even for films deposited at 5 Torr. The same series of growth conditions were then processed into solar cells, again comparing MgCl_2_ activation treatments at 410, 420, and 430 °C for each growth pressure. The performance parameters for these devices are shown in Figure 10, with the *JV* curves for the highest efficiency contact of each device shown in Figure 11. For all MgCl_2_ treatment temperatures, peak device efficiency is achieved at growth pressures below 100 Torr. Therefore, despite indications of reduced strain in the CdTe layer (Figure 9) with a slightly larger grain structure (Figure 7) for high-pressure growth, this has not translated to improved efficiency. While series resistance does decrease for higher pressure growth, this is offset by a rapid deterioration in shunt resistance and *V*_oc_, which leads to a decrease in efficiency. This reduced shunt resistance can be explained by incomplete coverage of CdTe on the SnO_2_ substrate shown in Figure 8, causing an increase in the fractional area of pinholes, which is visible to the naked eye. This confirms that CdTe growth is strongly influenced by the SnO_2_ substrate, leading to incomplete substrate coverage at high pressures resulting in a high density of pinholes which are not observed for growth on CdS.

The influence of the window layer on MgCl_2_ treatments is instantly apparent from Figure 10, with devices treated at 410 °C producing very low-efficiency devices regardless of growth pressure. This is in stark contrast to CdS/CdTe devices, where treatment at 410 °C produced the highest efficiencies. Although for SnO_2_/CdTe devices there is an initial improvement in all performance parameters with increased pressure up to 100 Torr for the 410 °C series, beyond this the open circuit voltage is decreased, limiting the maximum efficiency to 1.6%. These devices are primarily limited by a low fill factor, which can be seen in Figure 11a to result in an “S” shaped *JV* curve. This is commonly seen for CdTe junctions with MZO,^4,7^ and is typically attributed to poor electron extraction from the CdTe layer leading to charge accumulation. Increasing the treatment temperature to 420 °C improves device efficiency due to an increased short circuit current density and fill factor. Figure 11b shows a better diode response with intermittent and much less severe “S” shaped curves resulting in a dramatic reduction in series resistance and increased shunt resistance. There is a further increase in efficiency for devices treated at 430 °C due to increased fill factor caused by decreased series resistance. Further tests on higher temperature MgCl_2_ treatments indicate that there is no additional improvement above 430 °C, whereby devices become overtreated.

The “S” shaped curves observed for low treatment temperatures are common for CdTe devices and are typically attributed to a spike in the conduction band at the interface between the absorber and window layer as a result of a small electron affinity. While a small (Δ*E*_C_ < 0.1 eV) spike in the conduction band can be beneficial by reducing recombination at the interface, a large spike would result in a current blocking effect, which manifests as an “S” shape in *JV* curves such as those seen in Figure 11. This phenomenon is observed in a wide range of solar cell technologies^25^ and is more generally attributed to the presence of a charge transport barrier. The reported electron affinity of around 4.5 eV for bulk SnO_2_ matches well with that of CdTe; therefore, a flat band alignment would be expected.^26^ However, this is likely to be an overly simplistic approach. Predicting the band alignment at a heterojunction interface from literature values of bulk materials is notoriously challenging,^27,28^ and it is further influenced by the location of the Fermi level within both the window and absorber layers and defects near the interface. The reported work function of SnO_2_ varies significantly in the literature and shows strong sensitivity to processing conditions,^29,30^ and is further complicated by surface dipole effects which make an accurate measurement of the work function challenging; therefore, bulk literature values are not likely to accurately represent the conditions a real interface.^31^ However, removal of the “S” shaped *JV* curves upon high-temperature MgCl_2_ treatment suggests that the band alignment is improved by the removal of a charge transport barrier at the junction interface, resulting in a flatter conduction band alignment. The MgCl_2_ treatment is unlikely to have a substantial impact on the electron affinity of either layer, and capacitance–voltage (*CV*) measurements do not indicate significant variation in CdTe doping density for different treatment temperatures (Figure S2). This leaves either a change in the work function of the SnO_2_ layer, strain relaxation, or passivation of interfacial defects that cause a secondary barrier as the likely sources of the improvement.

**Figure 12:**
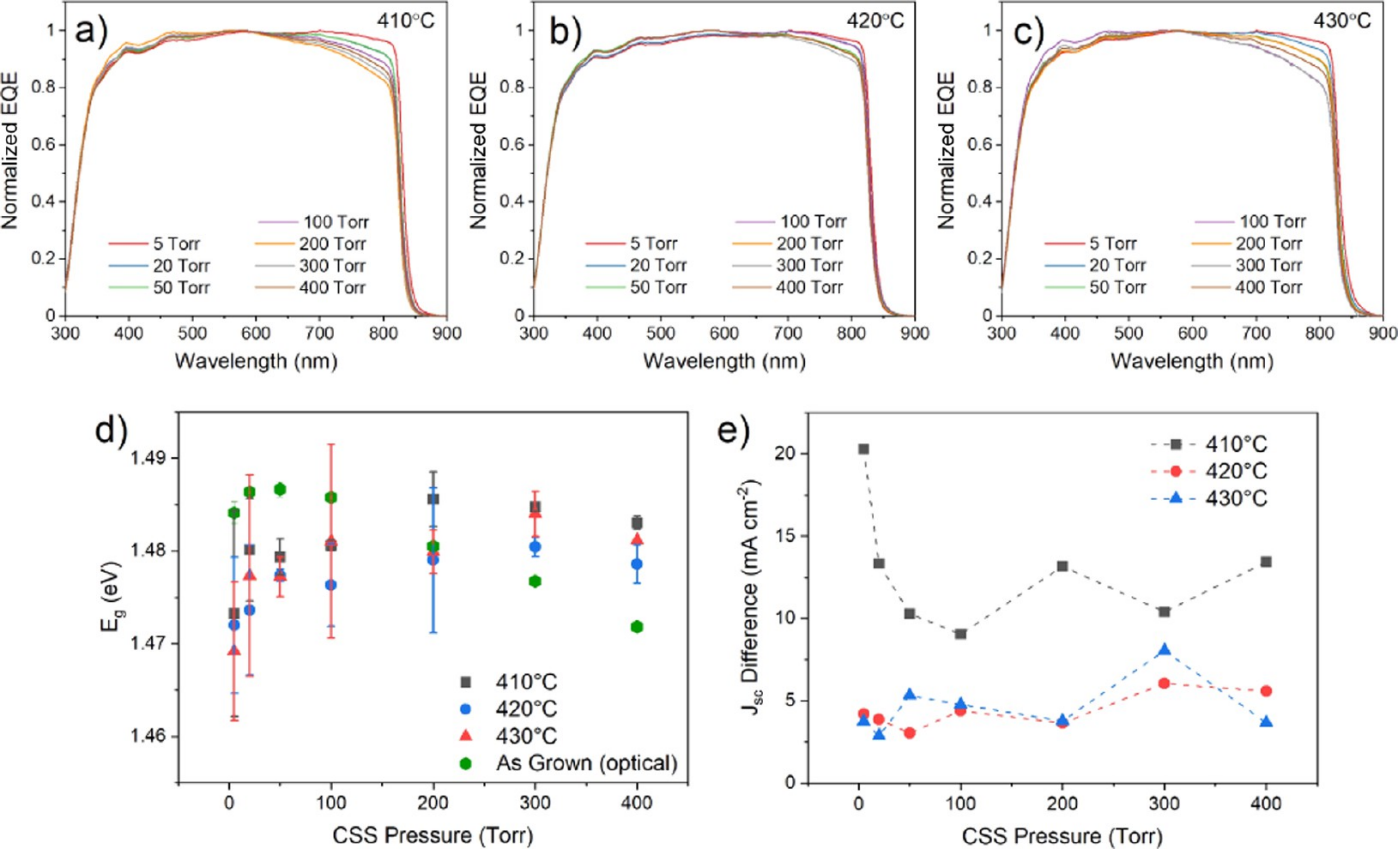
Normalized EQE spectra for devices grown on SnO_2_ under 5–400 Torr nitrogen and subject to MgCl_2_ treatment at 410 (a), 420 (b), and 430 °C (c) as well at the minimum absorber band gap taken from the intercept at long wavelength compared to the band gap of as-grown films estimated from optical absorption data using the Tauc method (d). Difference between short circuit current density measured from *JV* curves and integrated EQE curves (e).

Figure 12a–c shows the normalized EQE spectra for the highest efficiency cell from each device, corresponding to the *JV* curves shown in Figure 11. There is less variation between samples compared to CdS/CdTe devices with a square EQE shape for all measured cells. This is indicative of high collection efficiency across all wavelengths owing to the wide band gap of SnO_2_ in comparison to CdS. There is a noticeable variation in long wavelength collection (700–850 nm) that is most apparent for the 410 and 430 °C series; however, no systematic trend could be identified in either case, which might infer a strong sensitivity to small variations in carrier lifetime for these devices. The minimum absorber band gap was taken from the intercept of the CdTe absorption onset with the *x*-axis for each treatment temperature and is plotted as a function of growth pressure in Figure 12d, which also shows the band gap of untreated films determined via optical spectroscopy. This shows that the CdTe band gap is not affected by the MgCl_2_ treatment temperature and confirms that any improvement in band alignment inferred from Figure 11 is not due to changes in band structure on the absorber side. There is a small increase in band gap with increased growth pressure, although this is a minor effect with a maximum variation of 0.016 eV between all measured devices. Changes in the amount of strain at the interface could potentially alter the absorber band gap, and this was shown to have a dependence on growth pressure according to XRD measurements (Figure 9). The band gap of as-grown films, which were not processed into full devices, shows a much clearer trend compared to the MgCl_2_ treated films, with a maximum band gap at 50 Torr before decreasing linearly with growth pressure. This trend is subtly different than for the films that were processed into devices, and it remains unclear whether this is due to the difference in the method of measuring band gap or changes that occur upon MgCl_2_ treatment. Overall, the band gap values determined are larger than for the CdS/CdTe device due to the obvious absence of S/Te alloying.

Figure 12e shows the difference between the short circuit current density of devices measured directly from *JV* curves compared to integrating the EQE curves and accounting for the AM1.5G solar spectrum. In theory, both values should be identical; however, the data abovementioned shows that EQE measured *J*_sc_ values are consistently higher than from *JV* measurements. This difference can be explained by the different operating conditions under which the cells are measured, with EQE spectra collected in the dark and perturbed only by a small AC monochromatic light signal, whereas *JV* measurements are taken under AM1.5G light. The low injection conditions measured by EQE do not represent typical operating conditions for a solar cell, and therefore, *JV* measurements are considered to give a more accurate estimate of *J*_sc_. Nonetheless, the difference between these two measured values can give insight into the way in which photogenerated carriers can modify junction transport. Low short circuit current density compared to integrated EQE can indicate a barrier to photocurrent, whereby small current densities such as those observed during EQE measurements can pass such a barrier via thermionic emission, but high current densities observed under AM1.5G illumination cannot.^32^ Therefore, the difference between *J*_sc_ determined by *JV* and EQE measurements can give a rough indication of the size of the barrier. In this way, Figure 12e would indicate that MgCl_2_ treatment at 410 °C produces a large barrier for photogenerated carriers, which is alleviated to some extent by higher growth pressure. This is likely due to the long growth duration acting as an in situ anneal step. The *J*_sc_ difference for 420 and 430 °C MgCl_2_ treatments is further reduced and does not vary with treatment temperature or growth pressure, which suggests that the barrier height is lowered as much as possible. This interpretation would be consistent with the findings from Figure 11, whereby the “S” shaped *JV* curves result from misaligned conduction bands at the interface between SnO_2_ and CdTe. The exact nature of the band alignment at the interface which causes this barrier, as well as the mechanism by which it is alleviated by higher temperature MgCl_2_ treatment, remains unclear. However, given the improvement with growth pressure inferred from Figure 12e for the 410 °C series, which is consistent with indications of reduced strain in as-grown films (Figure 9), interfacial strain relaxation for high growth pressure or high-temperature MgCl_2_ treatment offers a plausible explanation.

The absence of hexagonal crystal facets and lack of systematic grain size change with growth pressure shown in SEM images, and areas of the exposed substrate shown from optical microscopy suggest that the growth of CdTe is substantially different on SnO_2_ in comparison to CdS. It can also be seen that SnO_2_ is an especially poor choice of substrate for high-pressure growth. The SnO_2_ layer, which is deposited via CVD, is likely to be rougher than the sputtered CdS layer, which may smooth out the underlying roughness of the substrate. This increased roughness could therefore alter the nucleation and growth of CdTe. Alternatively, differences in the lattice contact, bonding environment, and crystal structure of SnO_2_ substrates compared to CdS could contribute to the observed differences in the growth of CdTe films and the resulting grain structure.

The SnO_2_/CdTe device structure does not suffer from the intermixing issues found for the CdS/CdTe devices. However, the growth of the CdTe layer on SnO_2_ is highly strained due to mismatched lattice constants. Increasing the growth pressure relaxes this strain slightly, however the effect on the CdTe grain size is much weaker than for CdS substrates, and the larger grain structure is accompanied by progressively poorer substrate coverage providing shunting pathways. This means that despite the use of a more robust substrate, SnO_2_/CdTe devices remain limited to low growth pressures and fast deposition rates, producing small CdTe grains. However, these devices were not only tolerant to more aggressive chlorine treatments but also require ∼20 °C higher treatment temperatures compared to CdS/CdTe devices in order to operate effectively. A low treatment temperature, which was optimal for CdS/CdTe devices, causes a poor band alignment resulting in “S” shaped *JV* curves for SnO_2_/CdTe devices. This is alleviated to some extent by increasing the treatment temperature; however, it is likely that this device structure remains limited by poor band alignment in addition to strained growth of the CdTe layer.

### 3.3 SnO_2_/CdSe/CdTe Devices

It was shown in the previous section that the efficiency of SnO_2_/CdTe solar cells was limited by poor growth leading to incomplete coverage of the absorber layer, and nonideal band alignment indicated by “S” shaped *JV* curves. The use of higher temperatures than normal during MgCl_2_ treatment can improve the junction quality to some extent; however, a barrier to photocurrent is likely to persist regardless of processing conditions. To further modify the interface, a selenium-graded absorber layer can be incorporated which has been reported to improve carrier lifetime, increase *V*_bi_,^33,34^ and may also offer a route to an improved band alignment.

In this section, the deposited stack structure was SnO_2_/CdSe/CdTe which leads to alloying between CdTe and CdSe during CdTe deposition to produce a CdSe_*x*_Te_1–*x*_ layer at the near interface.^14^ The interfacial structure of the device is not completely different from a CdS/CdTe device structure; hence, it is instructive to again examine what influence the device processing has on structure and performance. Additionally, we may also expect the impact on the electron affinity of CdSe_*x*_Te_1–*x*_ due to variations in interdiffusion, which will subsequently have an impact on the band alignment with SnO_2_.

**Figure 13:**
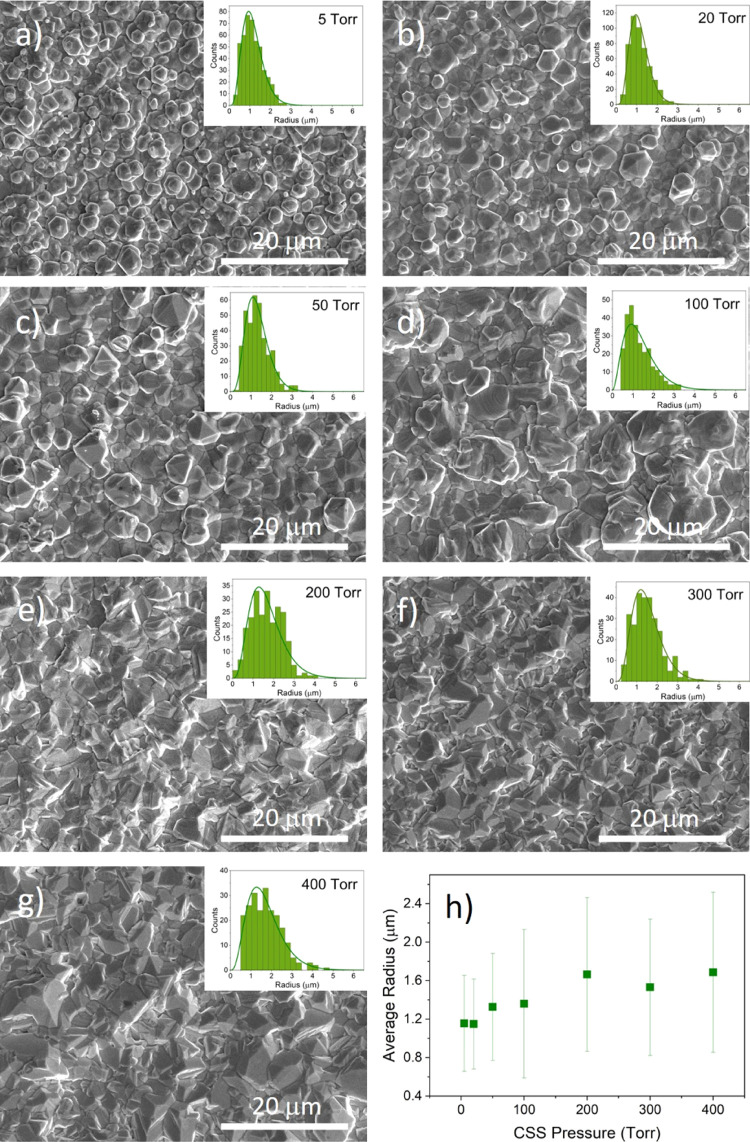
SEM images of the back surface of as-grown CdTe films deposited on CdSe at pressures between 5 and 400 Torr (a–g) with the grain size distribution shown inset, as well as the mean radius, plotted as a function of deposition pressure (h).

Figure 13a–g shows SEM images of the back surface of as-deposited CdTe films grown on SnO_2_/CdSe underlayers under nitrogen pressures between 5 and 400 Torr. The average grain radius is plotted as a function of growth pressure in Figure 13h. Low-pressure (i.e., 5 Torr) growth results in films with a hexagonal grain structure covering a more compact underlayer of tightly packed grains. The grain size is relatively uniform with an average radius of 1.2 μm. As the growth pressure is increased, the grain size increases slightly up to 200 Torr reaching an average radius of 1.6 μm, although many small grains remain and histograms become increasingly skewed. Average grain size remains constant at growth pressures above 200 Torr, while the grain shape becomes more irregular with sharp, well-defined crystal facets. The variation of morphology with growth pressure of CdTe films grown on CdSe is broadly similar to those grown on CdS (Figure 2), as might be expected given the similar crystal structure of both substrates. The grain size of films grown on CdSe substrates (Figure 13h) is smaller than for CdS substrates (Figure 2h), which is consistent with comparisons from EBSD measurements,^35^ and shows less variation with growth pressure. The average grain size for CdTe films grown on SnO_2_/CdSe substrates increases with growth pressure up to 200 Torr, which is not observed as clearly for growth directly onto SnO_2_. The grain size with SnO_2_/CdSe is also consistently lower for all growth pressures. The addition of CdSe between the SnO_2_ and CdTe layer, therefore, has a significant impact on the growth dynamics. However, SnO_2_/CdTe films grown at high pressures showed visibly poor substrate coverage with a high pinhole density, and there is no evidence of poor growth for these films. This suggests that depositing CdSe/CdTe bilayers is an effective method of improving the absorber film quality compared to a growing CdTe directly onto SnO_2_. This is distinct from the well established benefits of band gap grading a defect passivation.^33,36^ It is unclear whether the direct deposition of CdSe_*x*_Te_1–*x*_ would be affected by poor quality growth on SnO_2_ as found for CdTe, but a CdSe interlayer could be a similarly viable strategy to overcome this. While Kirkendall voiding is likely to be an issue for such a growth strategy, this could be mitigated by using a thinner CdSe layer to provide a suitable growth surface while limiting interdiffusion.

**Figure 14:**
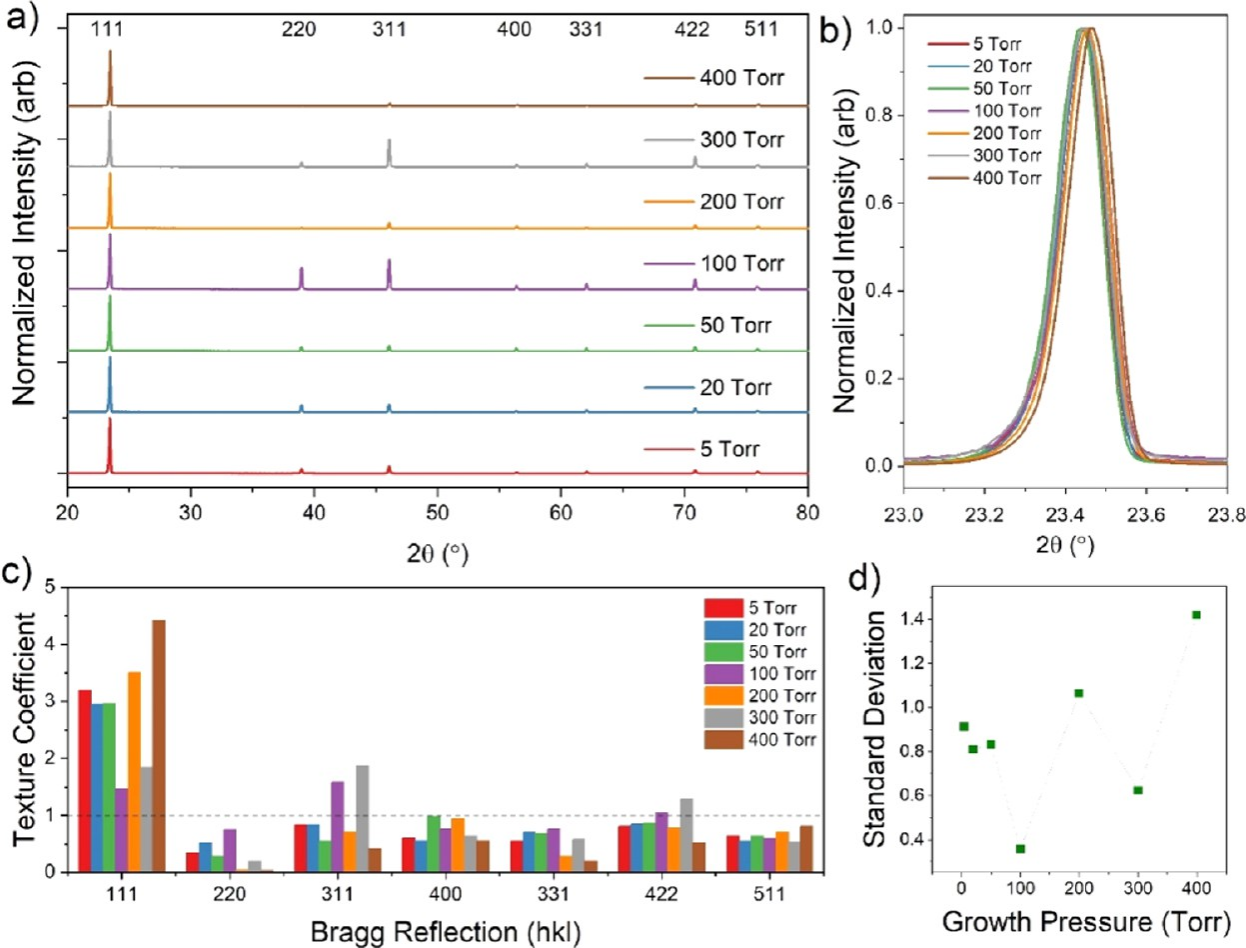
XRD data for 7 μm CdTe films grown on CdSe-coated substrates under varying pressure of nitrogen (a), with higher magnification of the 111 peak shown in (b). The texture coefficient for each Bragg reflection at each growth pressure is given in (c), and the standard deviation of the texture coefficients for each sample is in (d).

Figure 14 shows XRD patterns for the as-grown CdTe films deposited between 5 and 400 Torr onto sputtered CdSe. The mixing enthalpy for all compositions of CdSe_*x*_Te_1–*x*_ is lower than that of CdTe or CdSe at temperatures above ∼168 °C;^37^ therefore, the mixed alloy would be expected to readily form given the substrate temperature of 550 °C during CSS deposition. However, these diffraction patterns demonstrate that only a single CdTe phase is measured from the back surface with no sign of a CdSe or CdSe_*x*_Te_1–*x*_ phase. The dominant peak in each diffraction pattern is the 111 reflection centered around 23.45°, corresponding to a lattice constant of 6.57 Å which is larger than 6.48 Å expected for a powdered sample and literature values for bulk CdTe.^36^ This difference likely indicates residual tensile stress in the lattice, which has been found for all CSS-grown CdTe samples. There is some indication that the lattice constant decreases with increasing growth pressure as would be expected for increasing selenium content;^38^ however, this is less than the precision afforded by the resolution in diffraction angle and therefore represents only a minor difference. The lack of change with the addition of 100 nm CdSe is due to the thick (∼7 μm) CdTe film resulting in a very dilute alloy that does not extend throughout the sample in sufficient quantity to be detected, especially given the limited penetration of X-rays into the sample from the surface. It is not possible to determine from these measurements whether a selenium-rich phase exists at the front contact, although optical measurements shown in Figures S5–S7 imply that this is likely the case. Control over the selenium composition is especially critical here because CdSe_*x*_Te_1–*x*_ with *x* > 0.3 can crystalize in the wurtzite structure, which is harmful to photovoltaic applications.^36,39^ It is also noteworthy that the texture coefficient does not show a dependence on growth pressure for any of the Bragg peaks, with all films displaying a [111] preferential orientation.

**Figure 15:**
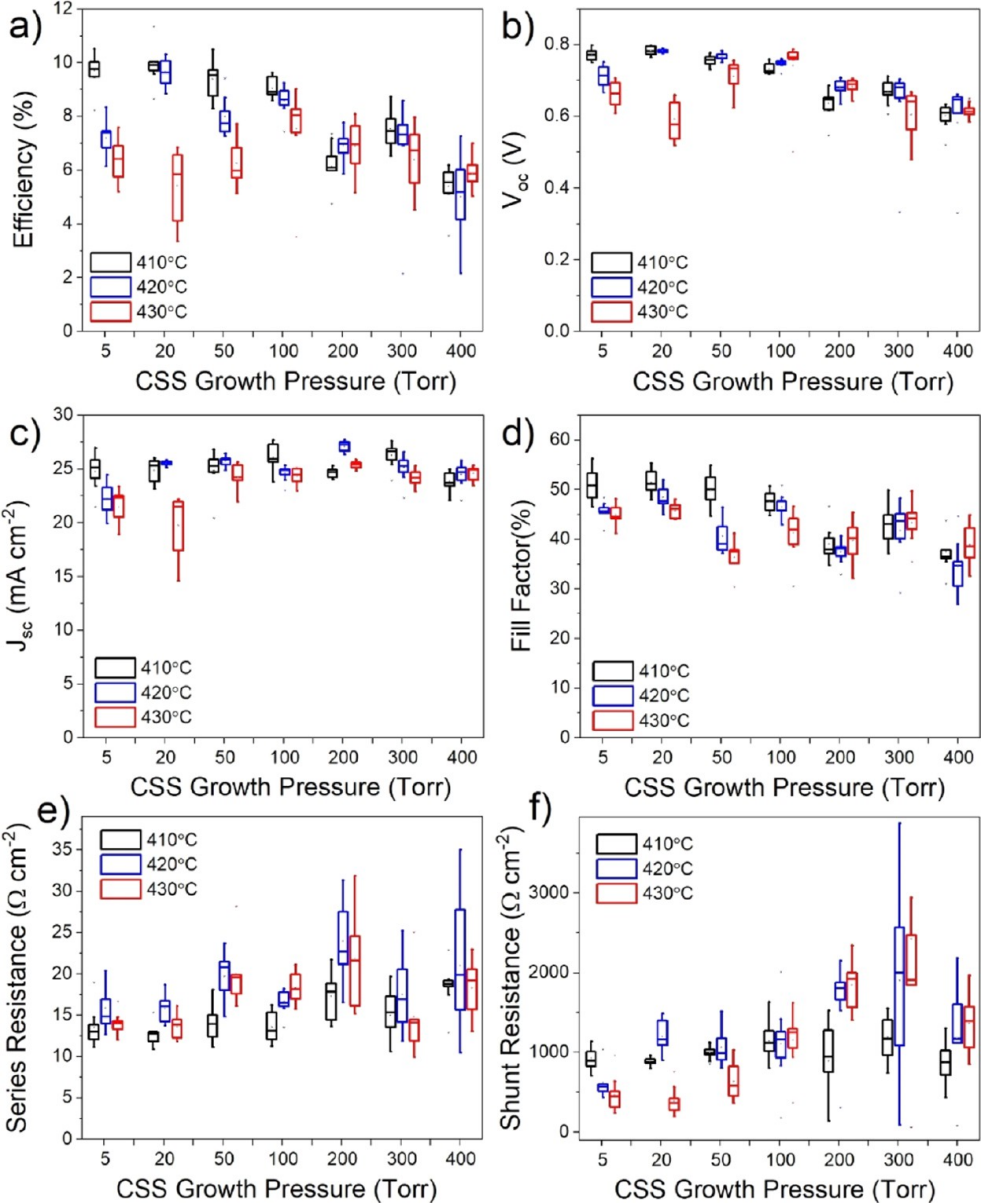
Box and whisker plots showing *JV* performance parameters for SnO_2_/CdSe_*x*_Te_1–*x*_ devices grown under 5–400 Torr of nitrogen, and activated at 410, 420, and 430 °C for each growth pressure. The box boundaries show the upper and lower quartiles with a horizontal line for the median value, and the range given by the whiskers. The efficiency (a), open circuit voltage (b), short circuit current density (c), fill factor (d), series resistance (e), and shunt resistance (f) are given as a function of growth pressure.

Performance parameters taken from *JV* curves of devices with varied growth pressure are shown in Figure 15 for MgCl_2_ activation temperatures of 410, 420, and 430 °C. In contrast to SnO_2_/CdTe devices, optimal processing conditions for SnO_2_/CdSe_*x*_Te_1–*x*_ devices involve lower temperature MgCl_2_ treatment, with efficiencies declining for higher temperatures similar to that observed for CdS/CdTe devices. All performance parameters contribute to this efficiency reduction, which is most noticeable for low growth pressures. Interdiffusion of CdSe into CSS grown CdTe is known to occur during chlorine activation as well as deposition^40^ in contrast to CdS, where sulfur diffusion primarily takes place during CdTe growth.^18^ Therefore, the effect of selenium redistribution due to MgCl_2_ treatment is expected to be most apparent for low growth pressures, as higher pressure growth is accompanied by longer deposition runs which will act to promote interdiffusion prior to the chloride treatment.^14^Figure 15 shows that higher pressure growth is seen to be detrimental to device efficiency due to a gradual reduction in both open circuit voltage and fill factor. However, despite this reduction, devices spanning a wide parameter space consisting of 21 processing combinations maintain reasonable performance, even for clearly overtreated cells that were subjected to several hours of high-temperature growth conditions. This contrasts with the CdS/CdTe and SnO_2_/CdTe devices described previously, where efficiency declines quickly outside of an optimal processing parameter window. Optimized SnO_2_/CdSe_*x*_Te_1–*x*_ devices reached higher efficiencies than the CdS/CdTe and SnO_2_/CdTe devices in this study. This is primarily due to an increase in *J*_sc_ afforded by a more transparent window layer compared to CdS while retaining high *V*_oc_ by improving the absorber interface with SnO_2_.

**Figure 16:**
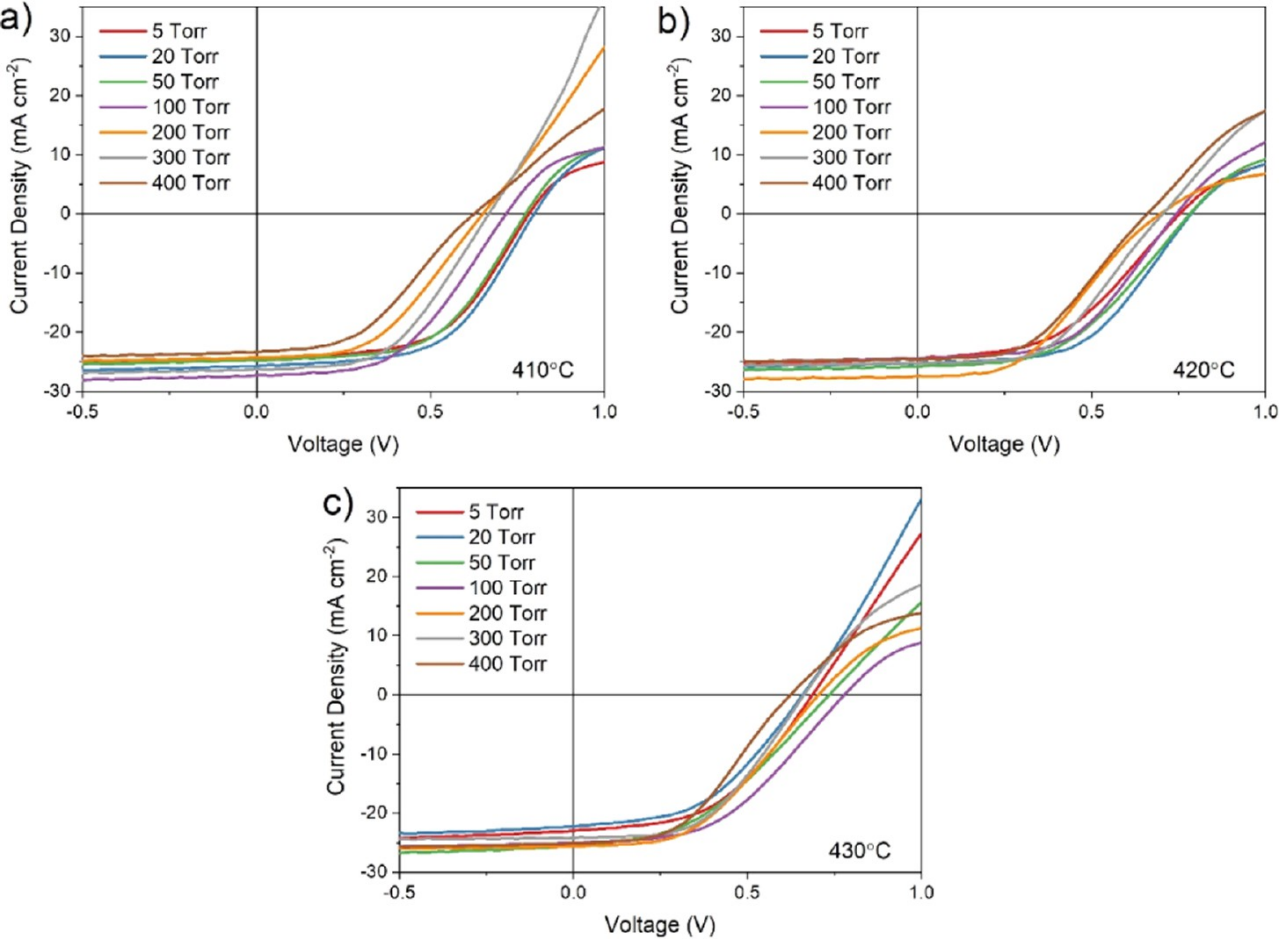
*JV* curves for the highest efficiency contact of SnO_2_/CdSe_*x*_Te_1–*x*_ devices grown under varied N_2_ pressure and treated at 410 (a), 420 (b), and 430 °C (c).

Figure 16 shows *JV* curves from the highest efficiency cell of each device. There is no sign of an “S” shape, which reduced the fill factor for SnO_2_/CdTe devices for any of the activation temperatures, indicating an improved band alignment at the front contact. While a small increase in the CBM of CdTe is expected with selenium alloying,^20^ this would be expected to increase the conduction band offset with SnO_2_ and, therefore, produce a larger barrier to the photocurrent. Therefore, the improved alignment indicated by the removal of “S” shaped *JV* curves, even for low MgCl_2_ temperature activation, is instead likely to result from elsewhere. This could be a reduced interfacial defect density due to selenium-induced passivation^36^ or less strained growth of CdTe on CdSe instead of directly onto SnO_2_. The uniformity of CdTe films has been improved by depositing CdSe onto SnO_2_-coated substrates prior to growth, with no evidence of pinholes and negligible above-band-gap light transmission for all samples, in contrast to when directly deposited onto SnO_2_. This allows for shunt resistance to be maintained or increased at higher growth pressures as shown in Figure 15f.

**Figure 17:**
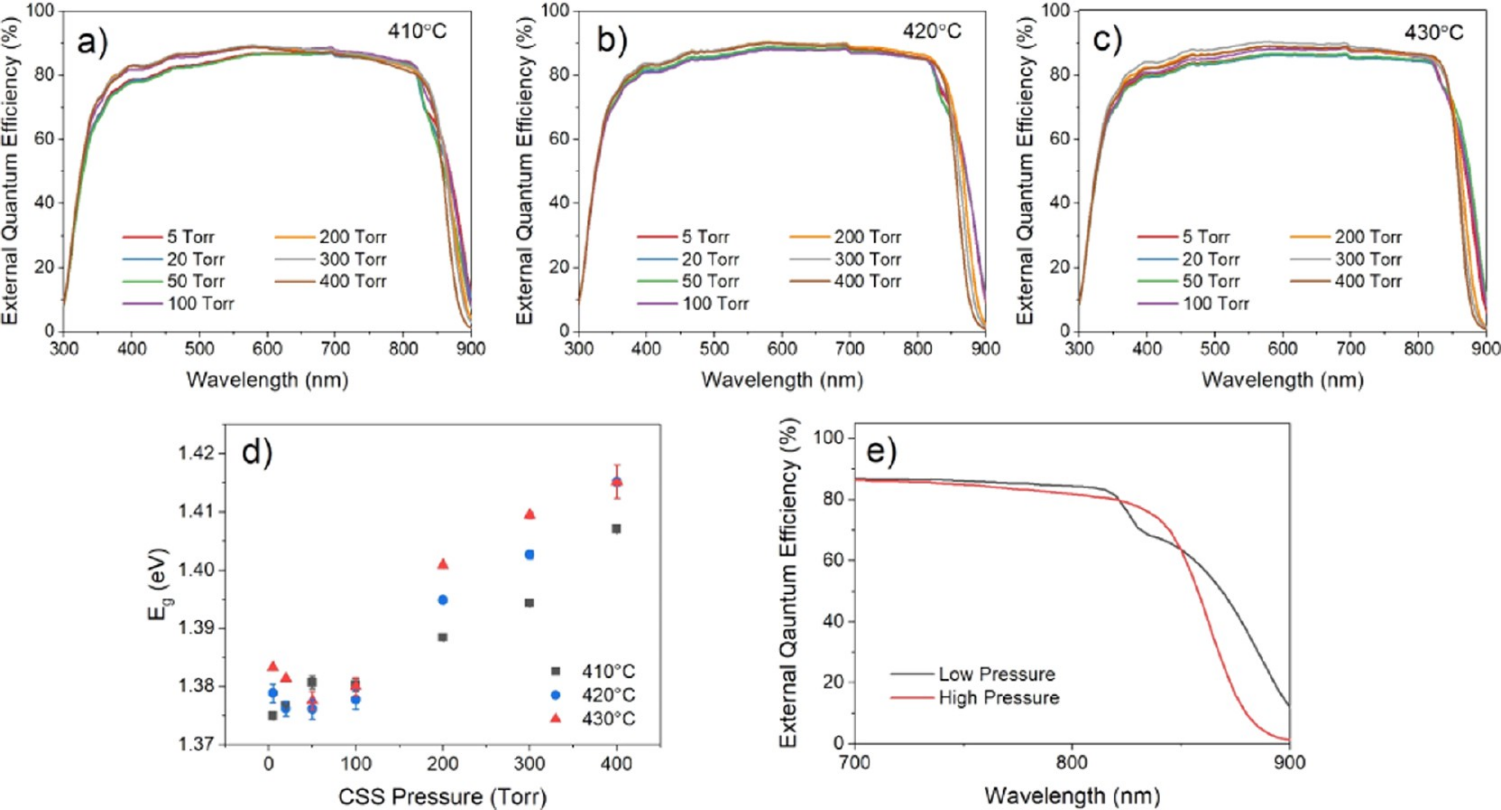
EQE spectra for devices grown on SnO_2_/CdSe under 5–400 Torr nitrogen and subject to MgCl_2_ treatment at 410 (a), 420 (b), and 430 °C (c) as well as the minimum absorber band gap (d). Example EQE curves demonstrating the qualitative differences between low (5 Torr) and high (400 Torr) growth pressure on the long wavelength region absorption shoulder €.

Figure 17a–c shows EQE measurements for the highest efficiency contacts from each device with varied growth pressure and MgCl_2_ activation temperature. While there is little systematic variation in the collection efficiency across most wavelengths, there is a noticeable shift in the long wavelength cut-off, which is commonly observed for selenium alloyed CdTe films.^41^ This results from the variation in the absorber band gap, which is estimated by linear extrapolation of the cut-off to the *x*-axis and shown as a function of pressure for the different treatment temperatures in Figure 17d. At low pressures, the band gap of the CdSe_*x*_Te_1–*x*_ layer is lower than both CdTe (∼1.45 eV) and CdSe (∼1.7 eV), reaching a minimum of 1.38 eV, which corresponds to a CdSe_*x*_Te_1–*x*_ layer with a composition of around *x* = 0.3.^37,38^ As the pressure is increased beyond 100 Torr, there is an increase in band gap as the selenium content becomes more dilute, with longer growth times encouraging its redistribution. The band gap increases linearly for each MgCl_2_ activation temperature up to a maximum of 1.41 eV. This remains lower than that of CdTe indicating that a selenium-rich region remains at the front contact for all devices despite the high mixing enthalpy and long growth times. This may be affected by the larger grain size for high-pressure growth restricting selenium diffusion, which has been shown to occur most readily along grain boundaries before migrating to the grain interior.^36^

Figure 17d shows the minimum absorber band gap estimated from linear extrapolation of the long wavelength EQE cutoff. The distribution of selenium is clearly influenced by both CSS deposition conditions and chlorine processing in contrast to CdS/CdTe devices, where sulfur distribution occurs primarily during CSS deposition.^18^ The shape of the EQE response at long wavelength varies with growth pressure for all activation temperatures, with the representative example shown in Figure 17e for clarity (the long wavelength region for all devices is shown in more detail in Figure S4). At low growth pressure, corresponding to a short growth duration, there are two separate absorption onsets from distinct CdSe_*x*_Te_1–*x*_ and CdTe layers around ∼830 and ∼870 nm, respectively. This may be explained in part by a difference in the thickness of the CdSe_*x*_Te_1–*x*_ layer as a function of growth pressure. Longer, high-pressure depositions would be expected to result in a relatively thick, more dilute alloy, which can fully absorb the incoming above-band-gap photons, whereas thinner, more Se-rich CdSe_*x*_Te_1–*x*_ layers may not. However, Figures S5–S7 indicate the existence of two distinct absorption edges for all samples irrespective of growth pressure; therefore, this cannot fully explain the observed changes toward the long wavelength EQE region. Instead, the selenium-rich alloys expected for low growth pressures likely cause the CdSe_*x*_Te_1–*x*_ layer to form a photo-inactive wurtzite phase at the front of the device, which does not contribute photocurrent.^42^ This would, therefore, result in parasitic absorption in the CdSe_*x*_Te_1–*x*_ layer for low growth pressures, which is alleviated for longer, higher pressure growth conditions, which encourage the formation of photoactive zincblende CdSe_*x*_Te_1–*x*_.

The addition of a CdSe layer between the SnO_2_ window layer and the CdTe absorber layer has been shown to have several beneficial effects. The growth of CdTe onto CdSe-coated SnO_2_ is much more favorable compared to deposition onto SnO_2_ directly, with no evidence of strained growth or poor substrate coverage at high growth pressures. There is a clear increase in grain size with higher growth pressures, although the grain size is slightly smaller than for CdS/CdTe devices, and there is no systematic effect on grain orientation. The CdSe and CdTe layers interdiffuse to form a graded CdSe_*x*_Te_1–*x*_ layer during the deposition process; therefore, higher growth pressures with slower deposition rates cause more intermixing, presumably altering the Se/Te grading profile. These SnO_2_/CdSe_*x*_Te_1–*x*_ devices show no evidence of the band alignment issues observed for SnO_2_/CdTe devices; however, it is not possible to determine whether the improved junction transport results from less strain in the absorber layer or from the different band gap of the CdSe_*x*_Te_1–*x*_ alloy. Despite this, reasonable device efficiencies were observed across a wide range of processing conditions, in contrast to CdS/CdTe and SnO_2_/CdTe devices.

## 4 Conclusions

This work provides an in-depth comparison of three device architectures linked to the device grain structure and postgrowth chloride treatment to determine the optimal processing conditions and compare the effectiveness of each. CdTe was initially grown on CdS substrates before being replaced with SnO_2_ as a more transparent window layer, and eventually incorporating a selenium-graded absorber layer by depositing CdTe onto a CdSe/SnO_2_ bilayer. For each structure, the absorber growth pressure was varied between 5 and 400 Torr, and the structural properties of the as-grown material were compared. These films were then processed into solar cells, using MgCl_2_ activation temperatures between 410 and 430 °C, which allows for a direct, statistical comparison of device performance across 63 processing conditions.

The CdS/CdTe device structure has been standard for over 40 years; however, is limited by a low band gap window layer which causes parasitic absorption. Although high-pressure growth was shown to increase the average CdTe grain size, the extended duration of the high-temperature deposition also results in excessive intermixing of the CdS and CdTe layers. High-temperature MgCl_2_ treatments resulted in reduced carrier concentration and poor device performance. Therefore, this device structure is limited by the thermal budget available during processing without causing excessive interdiffusion. SnO_2_/CdTe devices benefit from a more transparent window layer allowing more photons to reach the absorber layer and does not interdiffuse during high-temperature processing. However, the CdTe films grown directly onto SnO_2_ were found to be of poor quality. Strain in the CdTe layer is relaxed to some extent by increasing the growth pressure; however, this was found to also result in poor coverage, leaving areas of exposed substrate and offering shunting pathways. Notably, the grain size and texture of these CdTe films were not found to be strongly correlated with growth pressure as for CdS/CdTe films. Devices were also found to be very sensitive to the MgCl_2_ treatment temperature. Low-temperature (410 °C) treatment produced poor device efficiency due to “S” shaped *JV* curves, which severely limits the fill factor and indicates charge accumulation at the interface due to a transport barrier in the conduction band. This is alleviated by increasing the MgCl_2_ treatment temperature up to 430 °C, causing a substantial increase in efficiency. However, the *V*_oc_ of SnO_2_/CdTe devices remains lower than for CdS/CdTe indicating inferior junction quality, with no increase in *J*_sc_ despite the more transparent window layer. By depositing CdTe onto SnO_2_/CdSe bilayers, the growth surface is changed, while also having the effect of incorporating selenium into the absorber layer. This allows for improved substrate coverage in comparison to direct deposition onto SnO_2_, leading to uniform films without pinholes. Because the CdSe and CdTe layers readily intermix during film growth, a graded CdSe_*x*_Te_1–*x*_ layer is produced, which has a lower band gap than CdTe and forms a junction with SnO_2_. The smaller band gap allows for increased current collection, resulting in higher *J*_sc_, with *V*_oc_ similar to that of CdS/CdTe devices and higher than SnO_2_/CdTe devices. No evidence of a charge transport barrier was observed for any SnO_2_/CdSe_*x*_Te_1–*x*_ devices, suggesting an improved band alignment compared to SnO_2_/CdTe. While reasonable efficiencies were obtained over a wide parameter space with this device structure, interdiffusion occurs during both the absorber deposition and MgCl_2_ treatment, which is expected to significantly impact device performance. Therefore, using this approach, it was not possible to disentangle the effect of the selenium grading profile from other changes within the device which occur simultaneously, such as grain size and defect passivation.

The SnO_2_/CdSe_*x*_Te_1–*x*_ device structure combines a wide band gap window layer with a lower band gap absorber layer, allowing the *J*_sc_ of devices to be improved while retaining similar *V*_oc_ to the more traditional CdS/CdTe device structure. While some control over the microstructure of CdSe_*x*_Te_1–*x*_ is afforded by changing the growth pressure, the maximum grain size observed in this study remains below the film thickness, leading to a high density of grain boundaries within the absorber layer. However, further investigations into the effect of the window layer on the early stage nucleation and growth of CdTe would be beneficial in determining optimal processing parameters to produce large-grained, high-quality absorber layers. A large, well-oriented grain structure would result in fewer high-angle grain boundaries, especially those running parallel to the junction. The effect of substrate properties such as grain size, roughness, chemistry, pretreatments, and passivation layers also offer potential avenues of investigation.

The data underlying this study are openly available in The University of Liverpool Repository at datacat.liverpool.ac.uk/id/eprint/1790.

## Supporting Information Available

CdTe deposition times at varied growth pressures, CV data for each device structure, and optical data for CdSe_*x*_Te_1–*x*_ untreated films (PDF)
