# Supplementary material for: Interrelation of the CdTe Grain Size, Postgrowth Processing, and Window Layer Selection on Solar Cell Performance
Source: ACS Appl Mater Interfaces. 2022 Sep 9;14(37):42188–207. doi: 10.1021/acsami.2c07609 (PMC9501911; doi:10.1021/acsami.2c07609)
Supplement: Supplementary file 1 — am2c07609_si_001.pdf [file am2c07609_si_001.pdf]

Supporting Information –

Interrelation of CdTe grain size, post-growth processing and window layer selection on solar cell performance

Thomas. P. Shalvey [a], Heath Bagshaw [b] and Jonathan. D. Major [a,*]

[a] *Stephenson Institute for Renewable Energy, Department of Physics, University of Liverpool, Liverpool L69 7ZF, UK.*

[b] *SEM Shared Research Facility, School of Engineering, University of Liverpool, Liverpool, L69 3GL, UK.*

*Corresponding author

Email: jon.major@liverpool.ac.uk

Table S1: Variation of growth time for 7µm CdTe films deposited between 5 – 400 Torr

**Pressure (Torr)**	**Growth Rate (µm min^{-1})**	**Growth Time (min)**
5	1.60	4.4
20	0.55	13
50	0.31	22
100	0.19	37
200	0.07	98
300	0.05	138
400	0.04	162

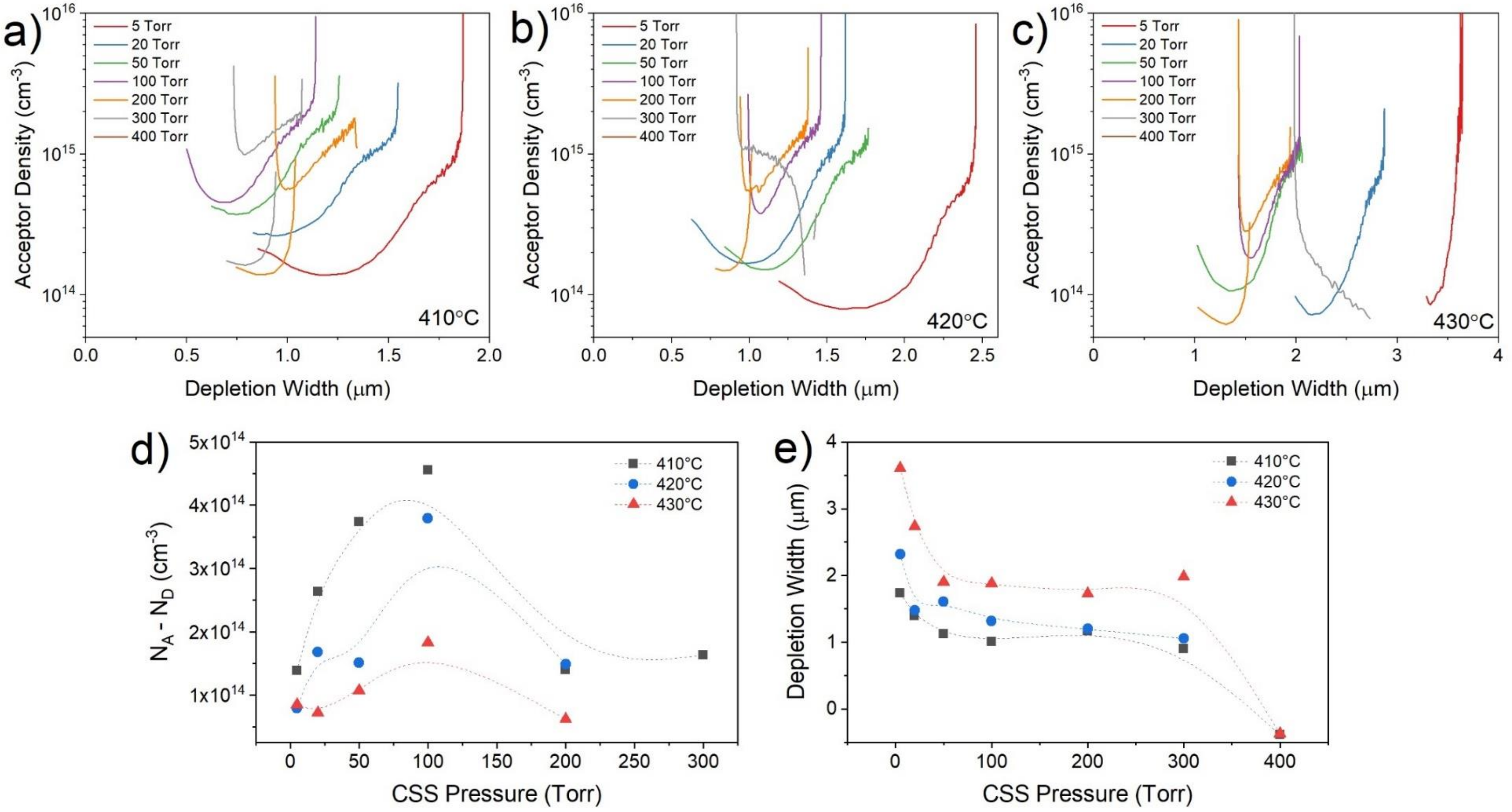

Figure S1: Acceptor density profiles for CdTe/CdS solar cells grown under 5 – 400 Torr nitrogen and $MgCl_2$ treated at 410°C (a), 420°C (b) and 430°C (c), with the acceptor density estimated from the minima of each curve shown as a function of pressure (d) and depletion width at zero bias shown in (e)

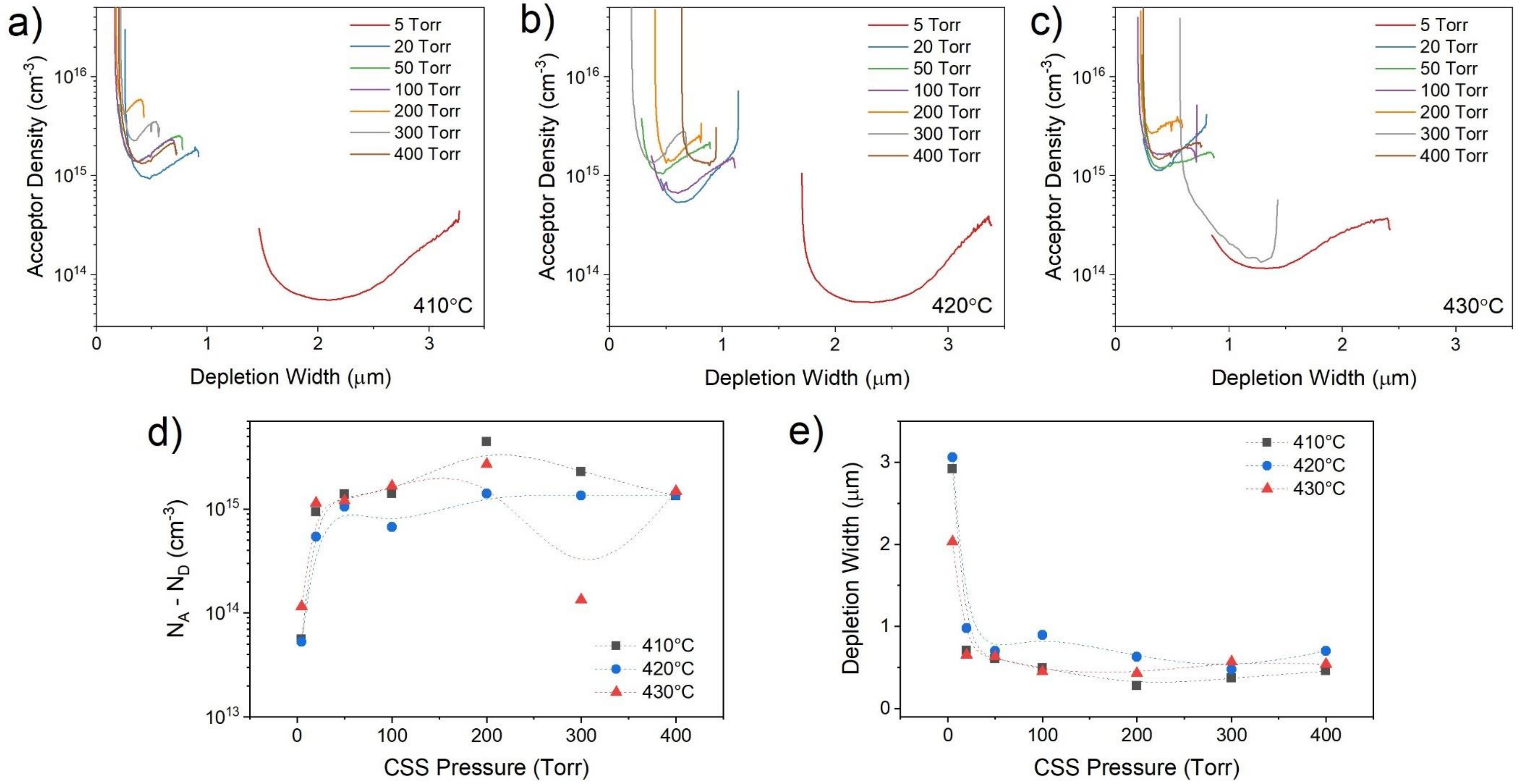

Figure S2: Acceptor density profiles for $CdTe/SnO_2$ solar cells grown under 5 – 400 Torr nitrogen and $MgCl_2$ treated at 410°C (a), 420°C (b) and 430°C (c), with the acceptor density estimated from the minima of each curve shown as a function of pressure (d) and depletion width at zero bias shown in (e)

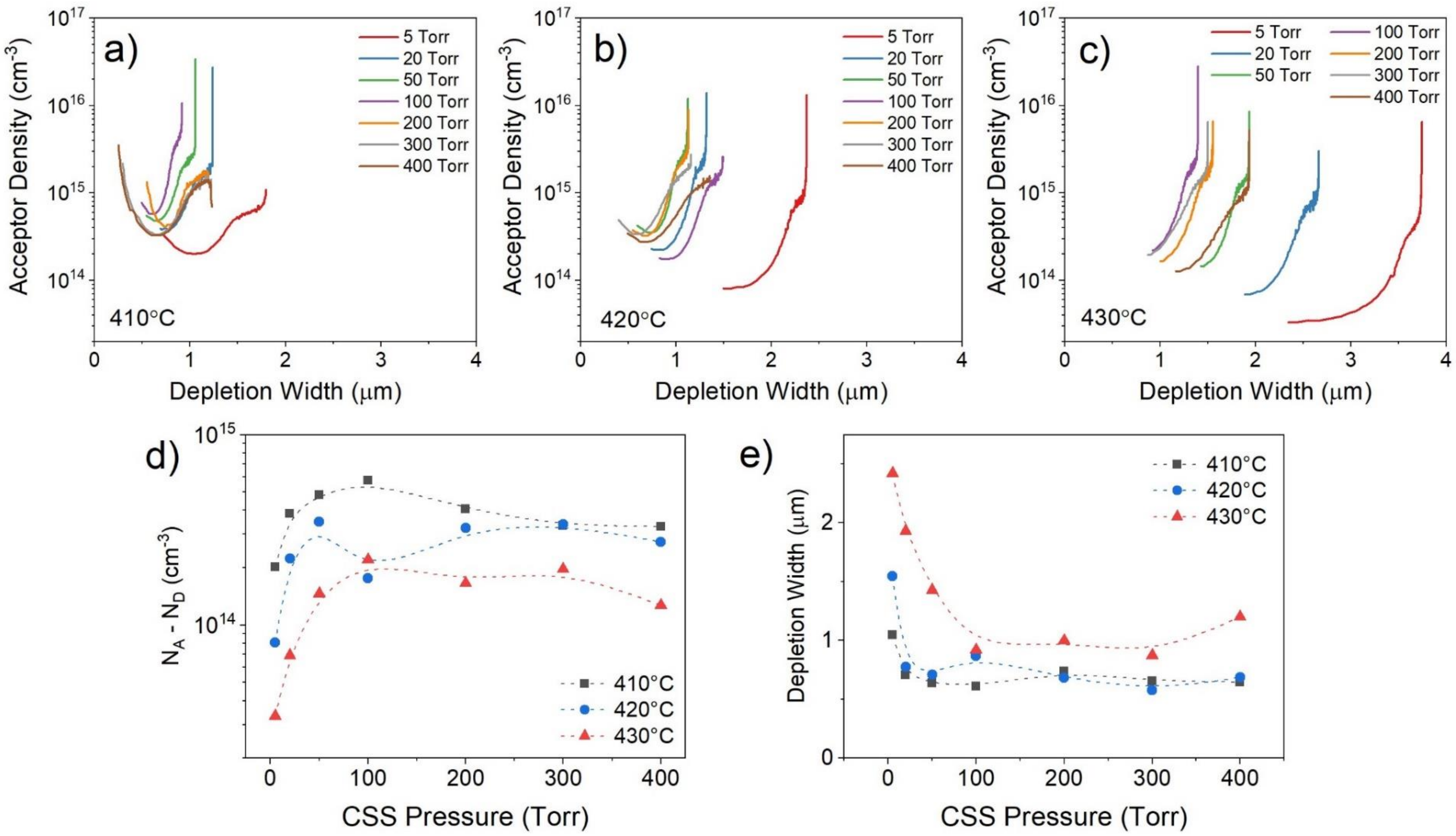

Figure S3: Acceptor density profiles for $SnO_2/CdSe_xTe_{1-x}$ solar cells grown under 5 – 400 Torr nitrogen and $MgCl_2$ treated at 410°C (a), 420°C (b) and 430°C (c), with the net acceptor density estimated from the minima of each curve shown as a function of pressure (d) and depletion width at zero bias shown in (e)

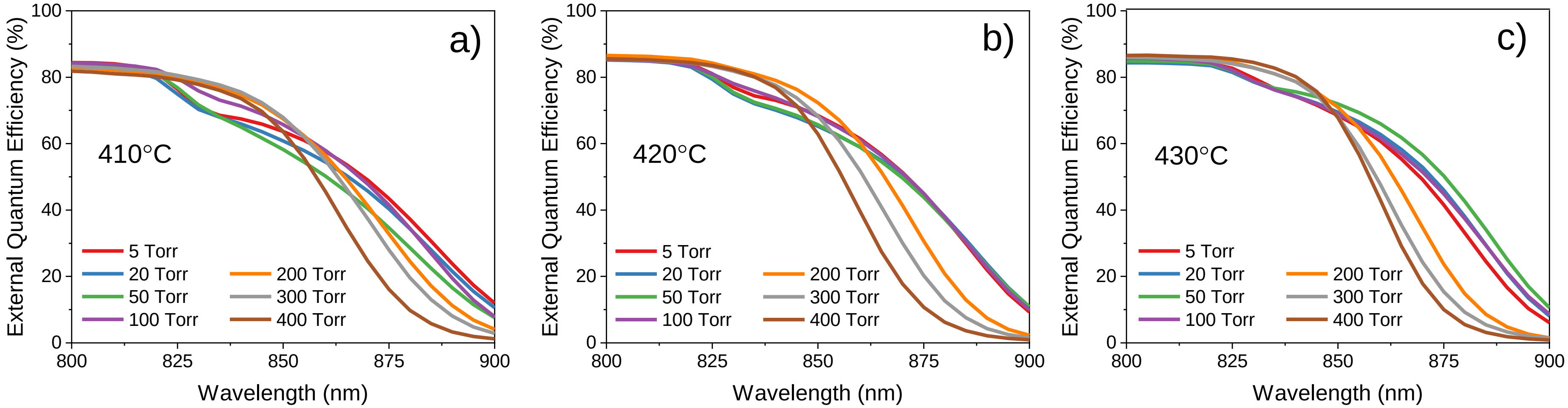

Figure S4: External quantum efficiency curves for $SnO_2/CdSe_xTe_{1-x}$ solar cells grown under 5 – 400 Torr nitrogen and $MgCl_2$ treated at 410°C (a), 420°C (b) and 430°C (c). This shows the long wavelength region in more detail whereby low-pressure growth results in the formation of two distinct collection edges, whilst higher pressure growth samples have only a single collection edge.

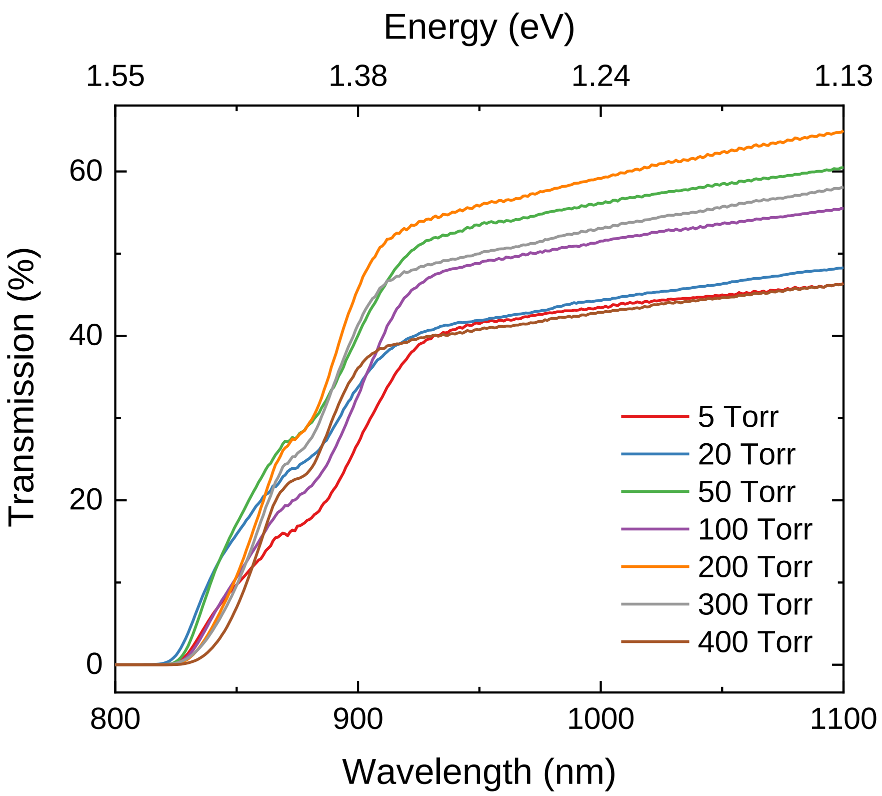

Figure S5: Transmission spectra for CdTe films deposited using CSS growth pressures between 5 – 400 Torr onto $CdSe/SnO_2$ substrates prior to $MgCl_2$ treatment.

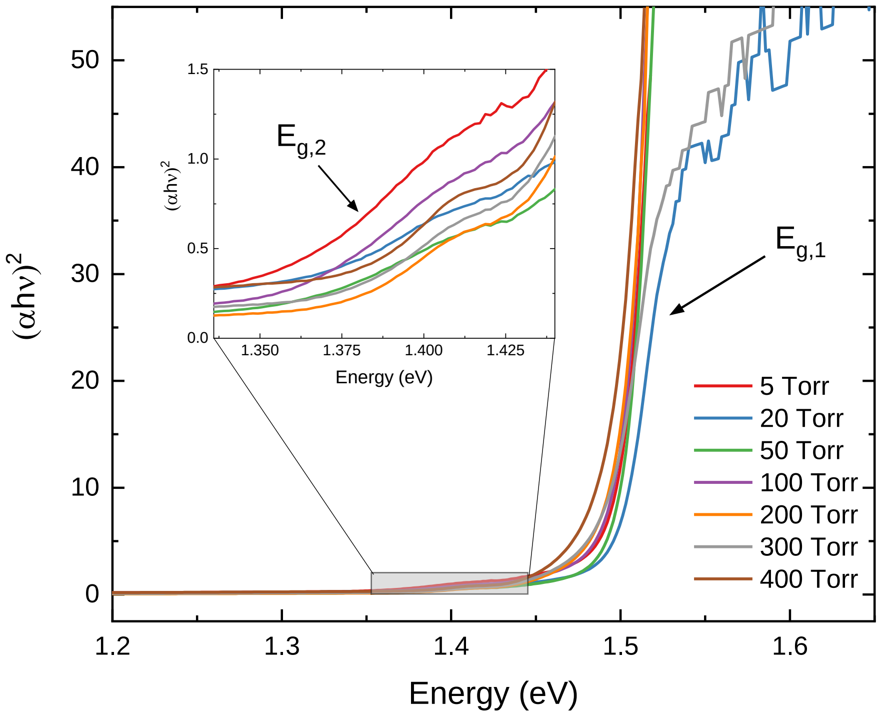

Figure S6: Tauc plot (n=2 for direct band gap) for CdTe films deposited using CSS growth pressures between 5 – 400 Torr onto $CdSe/SnO_2$ substrates prior to $MgCl_2$ treatment. Linear fitting of the dominant absorption edge towards the x-axis is used to determine the band gap $E_{g,1}$. Inset shows higher magnification of a secondary absorption edge, which is used to determine an associated secondary band gap $E_{g,2}$.

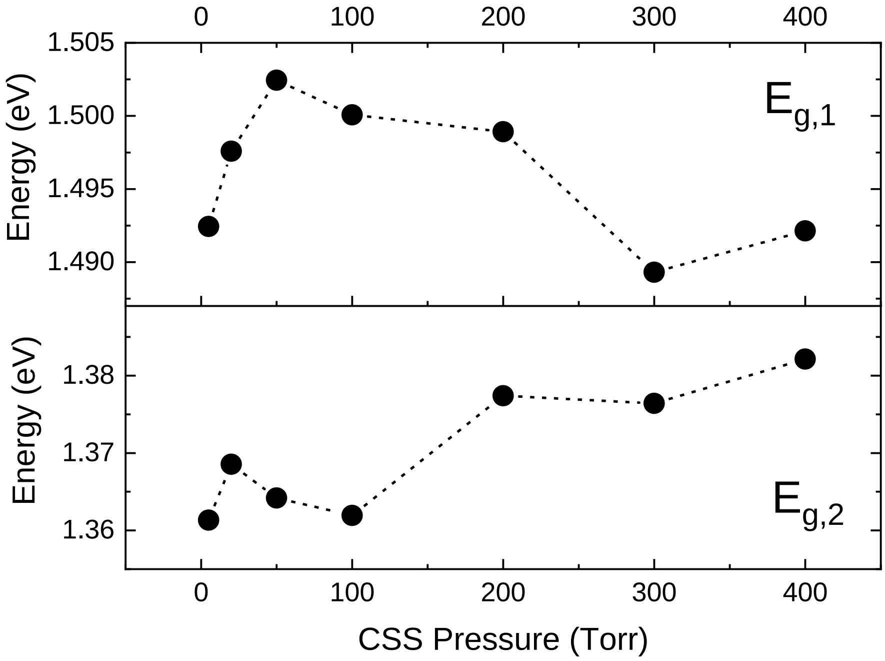

Figure S7: Band gaps extracted from the primary ($E_{g,1}$) and secondary ($E_{g,2}$) absorption edges from the Tauc plots shown in Figure S6 as a function of CSS growth pressure.

Figures S5-S7 show that CdTe films grown onto CdSe coated FTO substrates display two distinct absorption edges in all cases, including for high pressure CSS growth conditions which are exposed to elevated growth temperatures (>500°C) for several hours. Whilst it might be assumed that such processing conditions would lead to complete interdiffusion of the CdSe and CdTe layers to form a graded $CdSe_xTe_{1-x}$ layer, that does not appear to be the case here. The two absorption edges presumably arise from two distinct layers; one with a wider band gap ($E_{g,1}$) close to that expected for CdTe around 1.5eV, and another with a lower band gap ($E_{g,2}$) around ~1.37eV corresponding to an alloyed $CdSe_xTe_{1-x}$ layer which increases with growth pressure. The existence of two absorption edges is implied for low growth pressures from external quantum efficiency measurements in Figure 17e, but not for higher growth pressures. This discrepancy may arise from a selenium rich alloy for low growth pressures can result in a wurtzite $CdSe_xTe_{1-x}$ layer which is not photoactive [S1]. This layer therefore does not contribute photocurrent in EQE measurements, but does absorb light, therefore appearing as a shoulder in the long wavelength region. Higher growth pressures are likely to lead to more interdiffusion therefore more dilute, zinc-blende $CdSe_xTe_{1-x}$ layers, which are photoactive. These would be expected to lead to a single long wavelength EQE onset corresponding to the minimum absorber band gap, which is observed in Figure 17e.

[S1] Poplawsky, J., Guo, W., Paudel, N. et al. “Structural and compositional dependence of the $CdTe_xSe_{1-x}$ alloy layer photoactivity in CdTe-based solar cells” Nat Commun 7, 12537 (2016). https://doi.org/10.1038/ncomms12537
